# Supplementary material for: Role of Peptide Associations in Enhancing the Antimicrobial Activity of Adepantins: Comparative Molecular Dynamics Simulations and Design Assessments
Source: Int J Mol Sci. 2024 Nov 8;25(22):12009. doi: 10.3390/ijms252212009 (PMC11593906; doi:10.3390/ijms252212009)
Supplement: Supplementary file 1 [file ijms-25-12009-s001.zip › ijms-3247243-supplementary.pdf]

Supplementary materials:

Role of Peptide Associations in Enhancing the Antimicrobial Activity of Adepantins: Comparative Molecular Dynamics Simulations and Design Assessments

Matko Maleš [‡], Davor Juretić [§] and Larisa Zoranić [§]

[‡] *University of Split, Faculty of Maritime Studies, 21000 Split, Croatia.* mmales@pfst.hr

[§] *University of Split, Faculty of Science, Department of Physics, 21000 Split, Croatia,* davor.juretić@gmail.com

[§] *University of Split, Faculty of Science, Department of Physics, 21000 Split, Croatia,* larisaz@pmfst.hr

List of figures and tables:

Figure S1. 2D hydrophobic moments of peptides.

Figure S2. Center of mass distance from the membrane center of charged and hydrophobic residues of peptides.

Figure S3. Time evolution of secondary structures of peptides.

Figure S4. Density profiles of peptides and their charged and hydrophobic residues.

Figure S5. Number of clusters and number of peptides in largest cluster versus simulation time and cluster size distribution for twelve peptides interacting with membrane.

Figure S6. Characteristic states of peptides' interactions with a neutral POPC membrane.

Figure S7. Total number of contacts for each residue with other residues, calculated over the final 100 ns of simulations involving 12 peptides.

Figure S8. Distance matrices showing the smallest distances between residue pairs across all twelve peptides, calculated over the final 100 ns of simulations.

Figure S9. Visual representation of the clustering in AA-12 simulations.

Table S1. 3D Hydrophobic moments in simulations AA-1 case1.

Table S2. Electrostatic dipole moment vectors in simulations AA-1 case1.

Impact of the Adepantin studies

The impact of the design method and Adepantins in the research of antimicrobial peptides have been significant. Below, we briefly mention several studies as examples. In the study from 2012 [1] authors highlighted the innovative potential of Adepantins, grounded in the structure–activity relationships of naturally occurring AMPs. In the 2015 study [2] authors discussed the challenges in designing de novo structures based on physiochemical parameters (descriptors) and correlating them with biological activities through mathematical models, with Adepantins serving as exemplary designs. In 2019 study [3] authors delved into the D-descriptor as a hydrophobicity-based calculation method [4], explaining its function and method of calculation in more detail. A recent review from 2022 listed twelve promising peptide drug candidates, nine of which were designed by the Group of D.J., reflecting the impact of Adepantins' design principles [5]. The design of these nine peptides used our home-made algorithms, which we described in [4,6–9]. Furthermore, in 2023, authors emphasized the significance of sequence moments, noting that Adepantin-1, despite being derived from amphibian AMPs, does not resemble any natural AMP [10]. Additionally, the design of Adepantins has inspired the development of online servers predicting peptide function based on sequence. Examples include the design of Lys-rich peptides [11], the construction of the ENNAVIA server for predicting antiviral activity [12], the HAPPENN server for predicting the hemolytic activity of therapeutic peptides [13], and the ENNAACT server for predicting the antitumor activity [14].

Tools constructed in Prof. Davor Juretić laboratory

We have constructed several free-to-use online servers: Membrane Protein Secondary Structure Prediction Server - SPLIT 3.5 (http://split.pmfst.hr/split/), Membrane Protein Secondary Structure Prediction Server - SPLIT 4.0 (http://split4.pmfst.hr/split/4/), Therapeutic index estimator for frog-derived helical antimicrobial peptides (http://split4.pmfst.hr/split/dserv1/), Mutator algorithm for suggesting amino acid substitutions likely to improve the selectivity index of anuran or anuran-like peptides (http://split4.pmfst.hr/mutator/), MIC-Predictor server for predicting minimal inhibitory concentration (MIC) of Rana-box anuran peptides (http://splitbioinf.pmfst.hr/micpredictor/). Our goal was to provide powerful, fast, and user-friendly bioinformatics tools for those with minimal knowledge of rational design methods for constructing peptide antibiotics. The copy-paste functionality for inputting peptide sequences is so straightforward that even high school students can use it for in-silico antibiotic design projects. Additionally, we constructed the Database of Anuran Defense Peptides – DADP (http://split4.pmfst.hr/dadp/) [15]. This highly cited database helps users select parent peptides suitable for substitutions that enhance task-specific activities.

Our approach was firstly a) collecting an original database containing a certain class of peptides or proteins, secondly b) inventing some novel theoretical concept appropriate for the study of the database entries, thirdly c) constructing the user-friendly scientific server for free analysis of sequences by using new theoretical tool, and fourthly d) publishing the paper containing the link to the server, detailed description of all former steps and the superiority proofs regarding previous similar bioinformatic tools.

Details of CPP-Ad-1a construction

The CPP-Ad-1a was constructed using results from several online services, as explained in Juretić et al., 2022. The probability that the peptide is a cell-penetrating peptide was determined using the MLCPP server (old link: http://www.thegleelab.org/MLCPP/, new link: https://balalab-skku.org/mlcpp2/ [16]. We found antimicrobial peptide probabilities with the CAMPR3 Support Vector Machine algorithm on the server (http://www.camp.bicnirrh.res.in/predict [17]) and with AmpGram (http://biongram.biotech.uni.wroc.pl/AmpGram/ [18]). The ACPred server (http://codes.bio/acpred/ [19]) was used to classify peptides as anticancer (ACP) or non-anticancer (NACP) with a given probability but the mACPred server (old link: http://thegleelab.org/mACPpred/ [20], new link: https://balalab-skku.org/mACPpred2/ [21]) was used to find the probability of anticancer activity. For antiviral prediction we used servers ENNAVIA (https://research.timmons.eu/ennavia [12], FIRM-AVP (old link: https://msc-viz.emsl.pnnl.gov/AVPR, new link: https://github.com/pmartR/FIRM-AVP [22]) and Meta-iAVP (http://codes.bio/meta-iavp [23]). The iAMPpred and Antifungipept servers were used for the antifungal prediction (http://cabgrid.res.in:8080/amppred/server.php [24] and https://www.chemoinfolab.com/antifungal/ [25,26]). Results for the prediction of anti-inflammatory activity were collected by the AIPpred server (old link: http://www.thegleelab.org/AIPpred/ [27]), PreAIP server (http://kurata14.bio.kyutech.ac.jp/PreAIP/ [28]) and the score output of the AntiInflam server (old link: http://metagenomics.iiserb.ac.in/antiinflam/ [29]). The probability that the peptide has hemolytic activity was calculated by the HAPPENN server (https://research.timmons.eu/happenn [13]). The toxicity prediction was performed by the ToxinPred server (https://webs.iiitd.edu.in/raghava/toxinpred/ [30,31]). The amphiphilic character of the peptide was assessed by the SPLIT 3.5 server (http://split.djpept.com/split/ [32]). The predominantly disordered structure was indicated by the flDPnn server (http://biomine.cs.vcu.edu/servers/flDPnn/ [33]).

We used mean values of predicted probabilities for antimicrobial, anticancer, antiviral, and antifungal activity and mean value of predicted scores by AIPpred, PreAIP, and AntiInflam servers. Total score was calculated as: (CPP probability + mean antimicrobial probability + mean anti-cancer probability + mean antiviral probability + mean antifungal probability + mean anti-inflammatory score - (hemolytic activity probability + toxicity score)/2. The subtracted number was added as a positive reward for low toxicity. Among 176 peptides with calculated total score there were eight natural sequences, 40 peptides that were synthesized and tested in experiments, and 128 peptides designed by D.J. but never tested in experiments. We selected and ranked 20 peptides with the highest overall score for predicted multifunctional activity and low toxicity. All of them were designed by D.J. Eight top-scoring peptides are hybrids with the RRWFRRRRRR sequence or analogues of that sequence as the CPP part of their N or C terminal. That decapeptide was found in a natural sequence from the hypothetical protein OLQ14316.1 which belongs to the coral dinoflagellate symbiont *Symbiodinium microadriaticum*. Identical decapeptide R(122)RWFRRRRRR(131) can be also found in the sequence of an uncharacterized protein A0A5P1FK94 from the asparagus plant (*Asparagus officinalis*). The search motivation was a desire to find novel short nontoxic cell-penetrating peptides, by using the optimized penetrating analog RKKRWFRRRRPKWKK as a query [34]. High CPP activity and high uptake efficiency

were predicted for the RRWFRRRRRR decapeptide. Thus, it can potentially serve as a cargo carrier for antibiotics and anticancer molecules for different intracellular targets in human cells. For instance, intracellular bacterial pathogens are difficult to eradicate with conventional antibiotics, mainly due to the membrane permeability barrier [35], [36].

The hybrid peptide RRWFRRRRRR-Adepantin-1a is 19th in its overall score among 20 best peptides, and it has a perfect score for predicted antimicrobial and antiviral activity. Thus, it merits further theoretical and experimental investigations.

The links used in the development of Ad-1a and CPP-Ad-1a were accessed in August 2022 and all links were verified in July 2024. Note that both old (non-functional) and new (functional) links are provided for some of the servers.

Ad-1

Helix type: alpha
Full sequence:
GIGKHVGKALKGLKGLLKGLGES
Sequence length: 23 a.a.
Analysis window: 23 a.a.

$_1$**GIGKHVGKALKGLKGLLKGLGES**$_{23}$

Physico-chemical properties	Polar residues + GLY	Nonpolar residues
**Hydrophobicity <H>**	**Polar residues + GLY (n / %)**	**Nonpolar residues (n / %)**
0.275	15 / 65.22	8 / 34.78
**Hydrophobic moment <μH>**	**Uncharged residues + GLY**	**Aromatic residues**
0.596	HIS 1, SER 1, GLY 7	
**Net charge z**	**Charged residues**	**Special residues**
4	LYS 5, GLU 1,	CYS 0, PRO 0

Hydrophobic face : L A I L L V L L

Manual mutation GA mutation

Click to enlarge

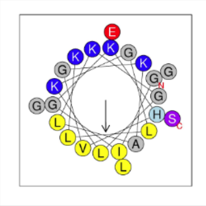

Data file (in txt format): Data.txt

Ad-1a

Helix type: alpha
Full sequence:
GIKKAVGKALKGLKGLLKALGES
Sequence length: 23 a.a.
Analysis window: 23 a.a.

$_1$**GIKKAVGKALKGLKGLLKALGES**$_{23}$

Physico-chemical properties	Polar residues + GLY	Nonpolar residues
**Hydrophobicity <H>**	**Polar residues + GLY (n / %)**	**Nonpolar residues (n / %)**
0.253	13 / 56.52	10 / 43.48
**Hydrophobic moment <μH>**	**Uncharged residues + GLY**	**Aromatic residues**
0.592	SER 1, GLY 5	
**Net charge z**	**Charged residues**	**Special residues**
5	LYS 6, GLU 1,	CYS 0, PRO 0

Hydrophobic face : L A I L L V L L

Manual mutation GA mutation

Click to enlarge

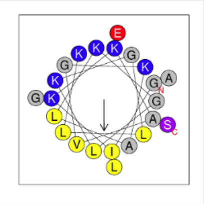

Data file (in txt format): Data.txt

CPP-Ad-1a

Helix type: alpha
Full sequence:
RRWFRRRRRRGIKKAVGKALKGLKGLLKALGES
Sequence length: 33 a.a.
Analysis window: 33 a.a.

$_1$**RRWFRRRRRRGIKKAVGKALKGLKGLLKALGES**$_{33}$

Physico-chemical properties	Polar residues + GLY	Nonpolar residues
**Hydrophobicity <H>**	**Polar residues + GLY (n / %)**	**Nonpolar residues (n / %)**
0.054	21 / 63.64	12 / 36.36
**Hydrophobic moment <μH>**	**Uncharged residues + GLY**	**Aromatic residues**
0.319	SER 1, GLY 5	TRP 1, PHE 1,
**Net charge z**	**Charged residues**	**Special residues**
13	LYS 6, ARG 8, GLU 1,	CYS 0, PRO 0
	**Hydrophobic face :** none	

Manual mutation GA mutation

Click to enlarge

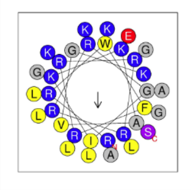

Data file (in txt format): Data.txt

CPP

Helix type: alpha
Full sequence:
RRWFRRRRRR
Sequence length: 10 a.a.
Analysis window: 10 a.a.

$_1$**RRWFRRRRRR**$_{10}$

Physico-chemical properties	Polar residues + GLY	Nonpolar residues
**Hydrophobicity <H>**	**Polar residues + GLY (n / %)**	**Nonpolar residues (n / %)**
-0.404	8 / 80.00	2 / 20.00
**Hydrophobic moment <μH>**	**Uncharged residues + GLY**	**Aromatic residues**
0.469	GLY 0	TRP 1, PHE 1,
**Net charge z**	**Charged residues**	**Special residues**
8	ARG 8,	CYS 0, PRO 0
	**Hydrophobic face :** none	

Manual mutation GA mutation

Click to enlarge

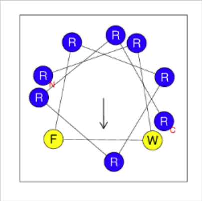

Data file (in txt format): Data.txt

Figure S1. 2D hydrophobic moments calculated by Heliquest server (https://heliquest.ipmc.cnrs.fr/cgi-bin/ComputParams.py)

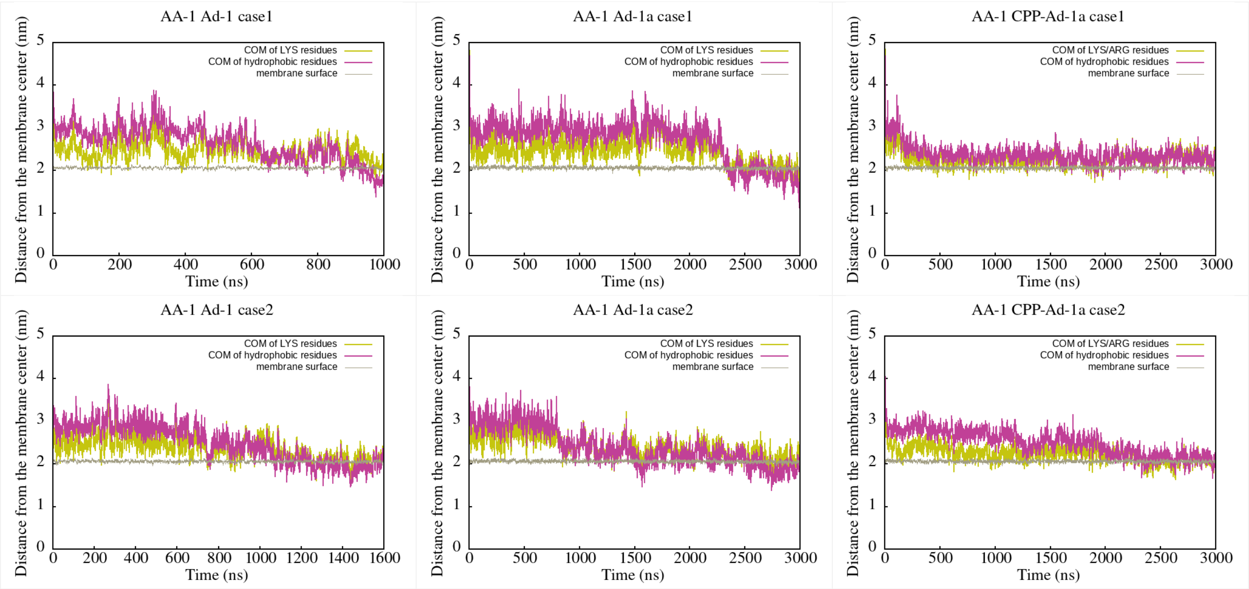

Figure S2a. Center of mass distance from the membrane center of charged (yellow) and hydrophobic (magenta) residues for Adepantin-1 (**left column**), Adepantin-1a (**middle column**) and CPP-Adepantin-1a (**right column**) in AA-1 simulations with **POPE:POPG membrane**.

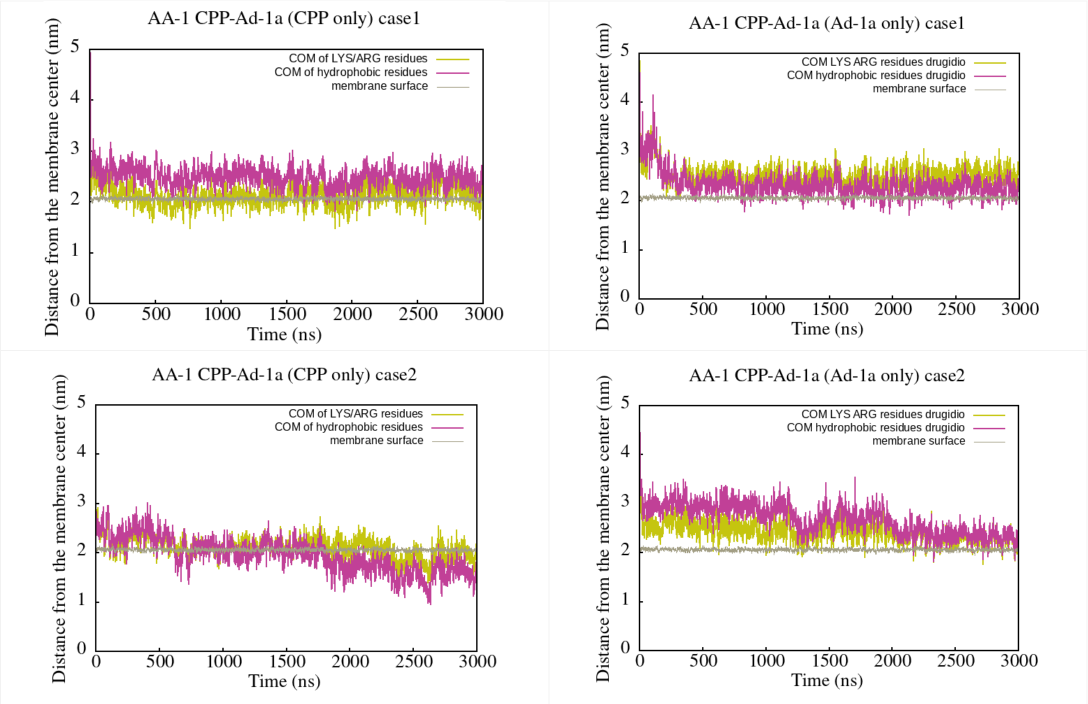

Figure S2b. Center of mass distance from the membrane center of charged (yellow) and hydrophobic (magenta) residues for CPP part only (amino acids 1-10) (**left column**) and Ad1a part only (amino acids 11-33) (**right column**) in AA-1 CPP-Ad1a simulations with **POPE:POPG membrane**.

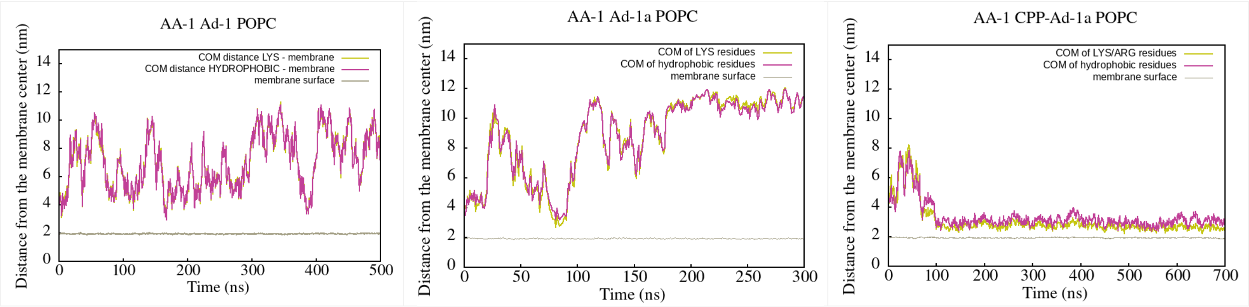

Figure S2c. Center of mass distance from the membrane center of charged (yellow) and hydrophobic (magenta) residues for Adepantin-1 (**left column**), Adepantin-1a (**middle column**) and CPP-Adepantin-1a (**right column**) in AA-1 simulations with **POPC membrane**.

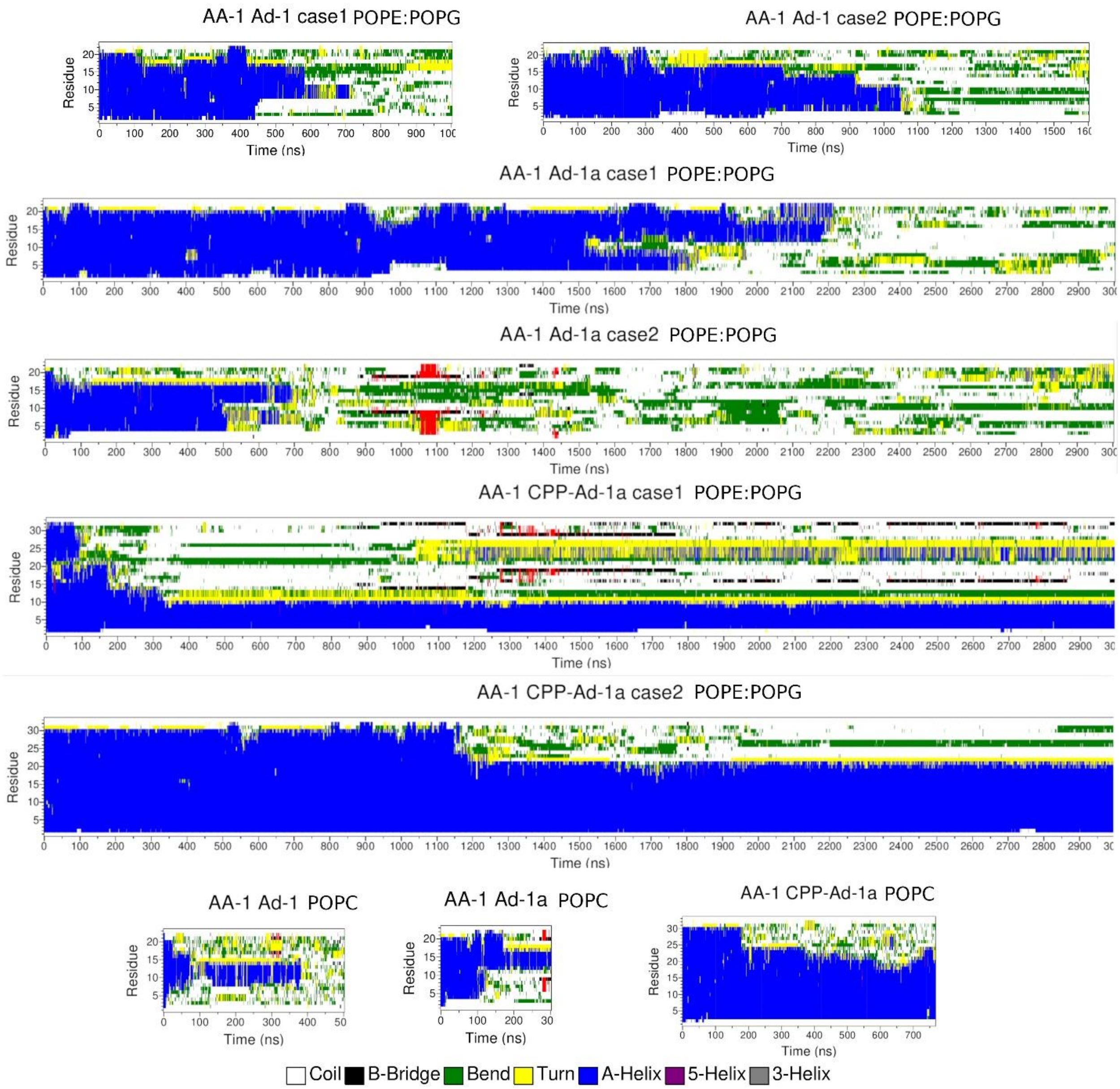

Figure S3a. Time evolution of secondary structures of peptides in AA-1 simulations, obtained using the DSSP program [37,38].

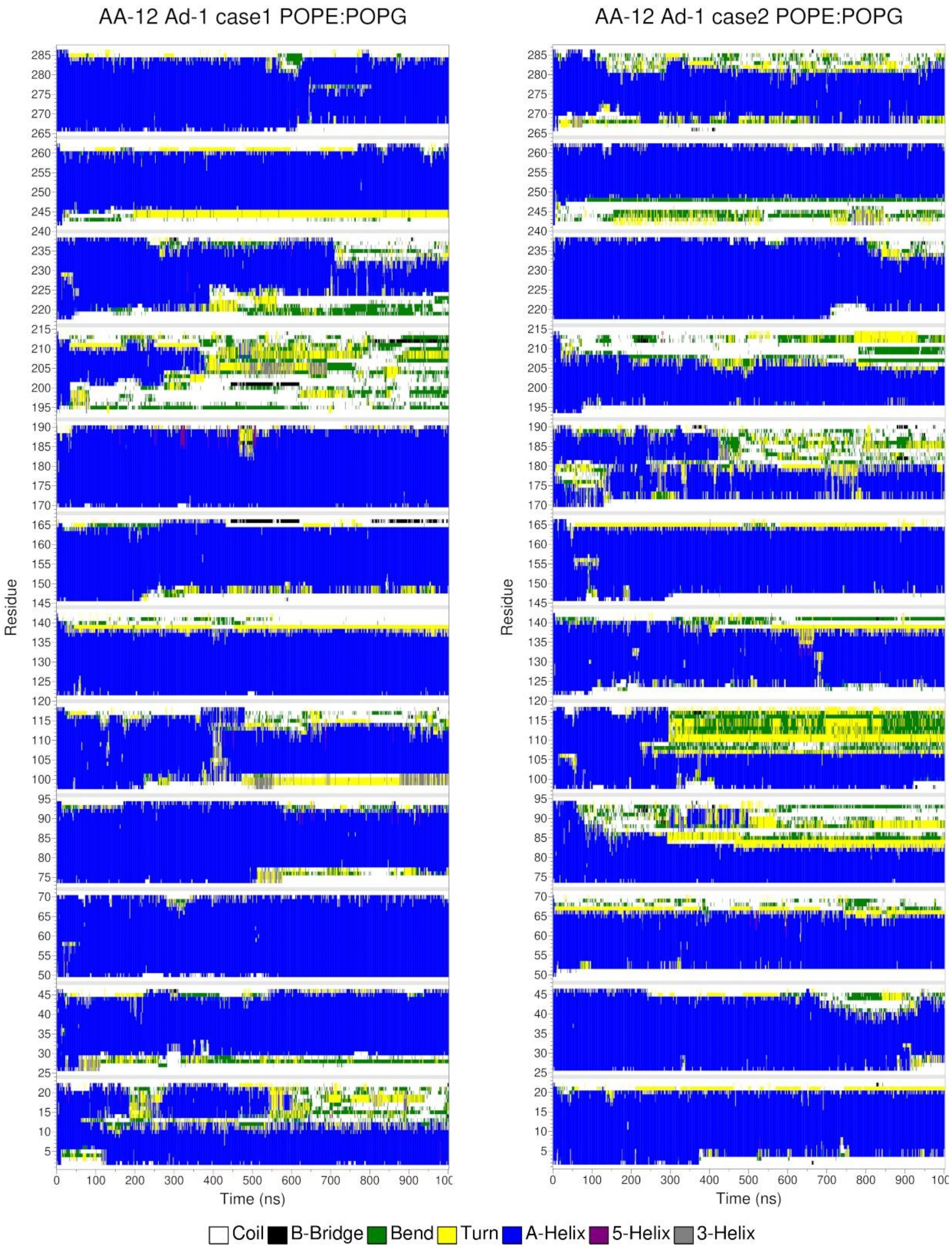

Figure S3b. Time evolution of secondary structures of peptides in AA-12 Ad-1 simulations, obtained using the DSSP program [37,38].

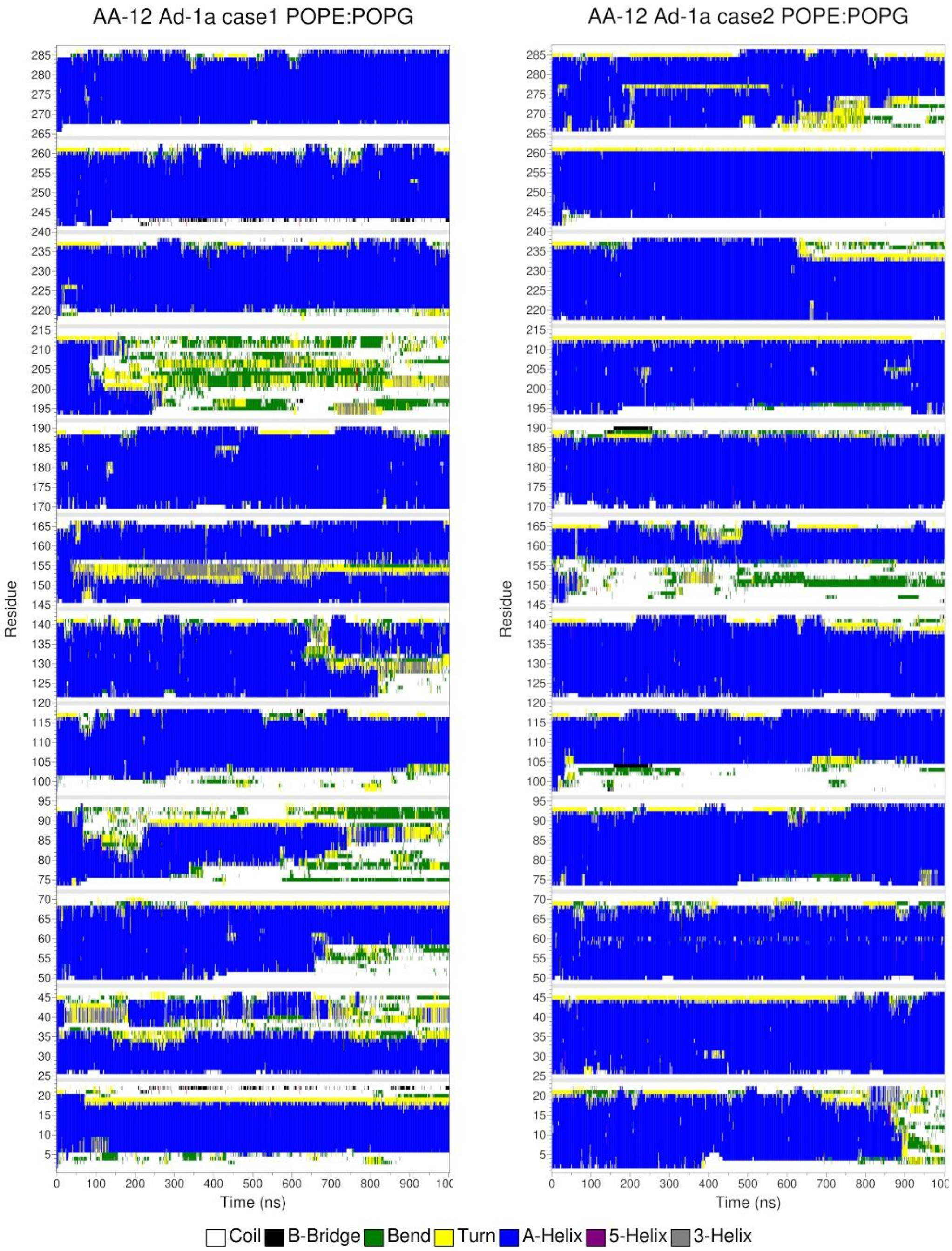

Figure S3c. Time evolution of secondary structures of peptides in AA-12 Ad-1a simulations, obtained using the DSSP program [37,38].

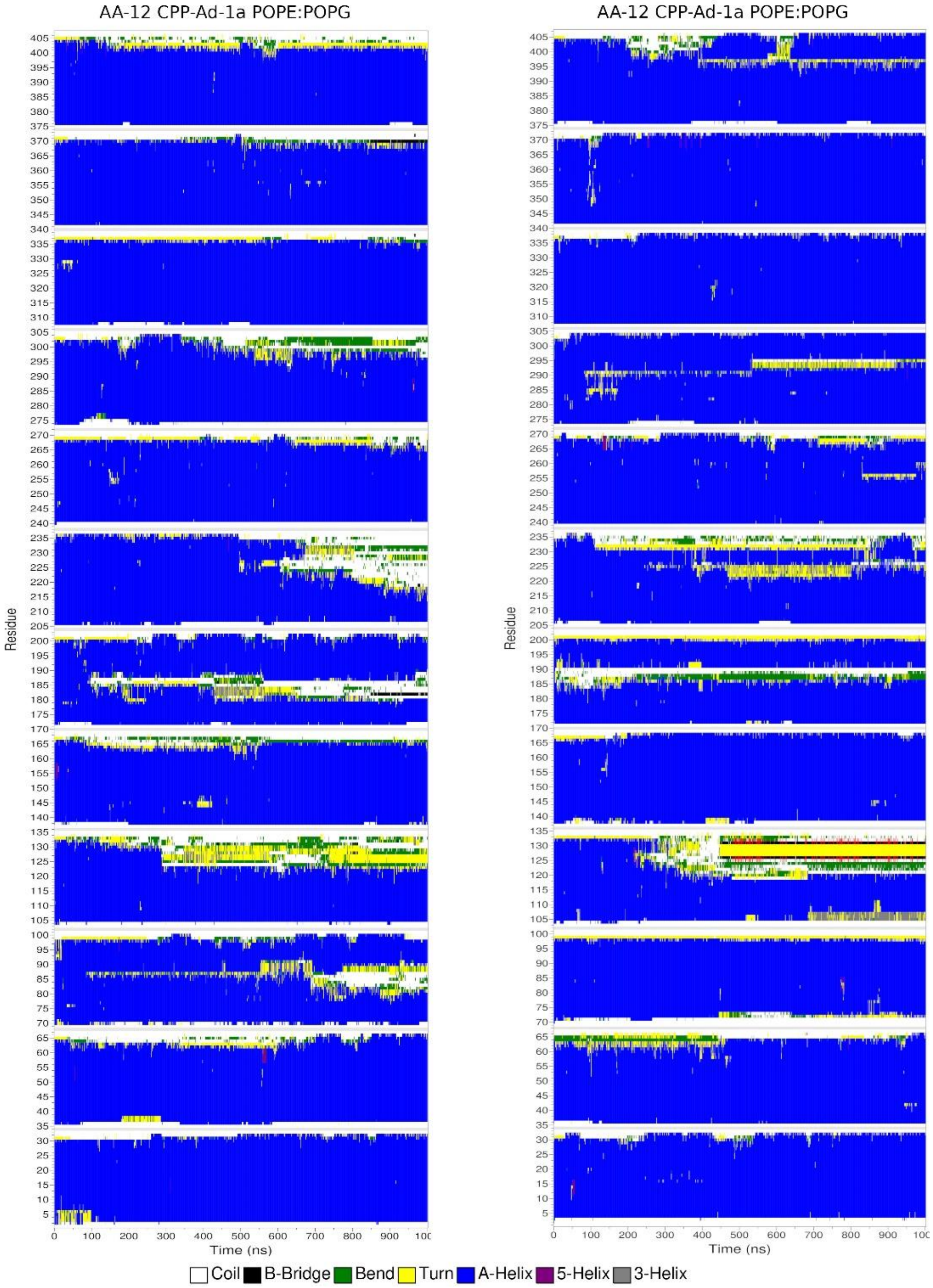

Figure S3d. Time evolution of secondary structures of peptides in AA-12 CPP-Ad-1a simulations, obtained using the DSSP program [37,38].

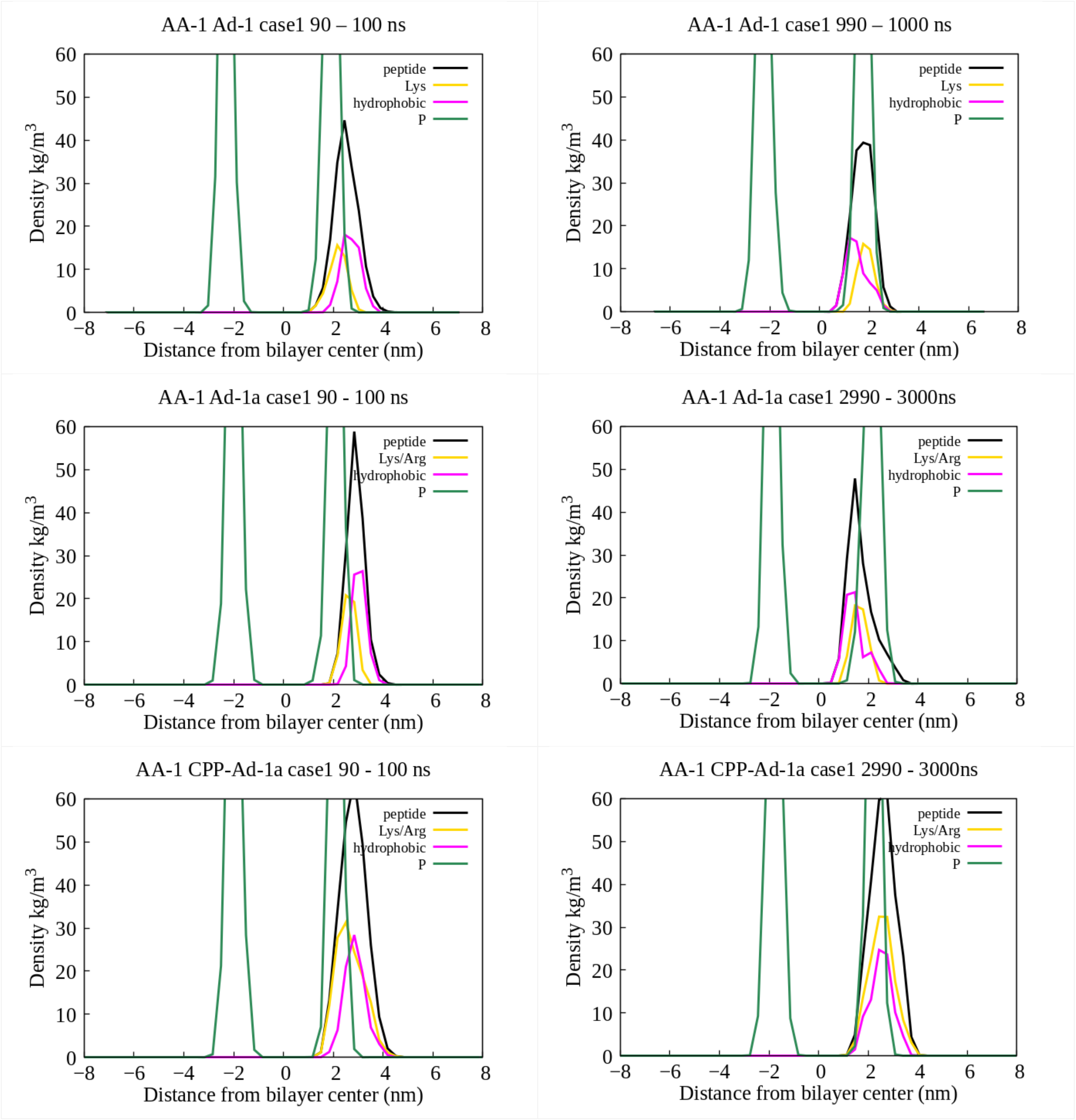

Figure S4a. Density profiles calculated with Gromacs *density* tool of hydrophobic (magenta) and positively charged LYS or ARG residues (yellow) in AA-1 simulations (**case 1**), averaged over denoted time intervals. **1st row:** Ad-1, **2nd row:** Ad-1a, **3rd row:** CPP-Ad-1a. **Left column:** At early stages of interaction of the peptide with the membrane (90 – 100 ns interval), peptide is oriented in such a way that positively charged Lysine residues are closer to the anionic upper leaflet surface than hydrophobic residues, supporting the role of electrostatic interactions in initial binding. **Right column:** at later stages (last stages in simulations) hydrophobic residues are closer to the central plane of the membrane revealing the turnover of the peptide over time and importance of hydrophobic interactions with carbohydrate lipid chains after initial electrostatic binding.

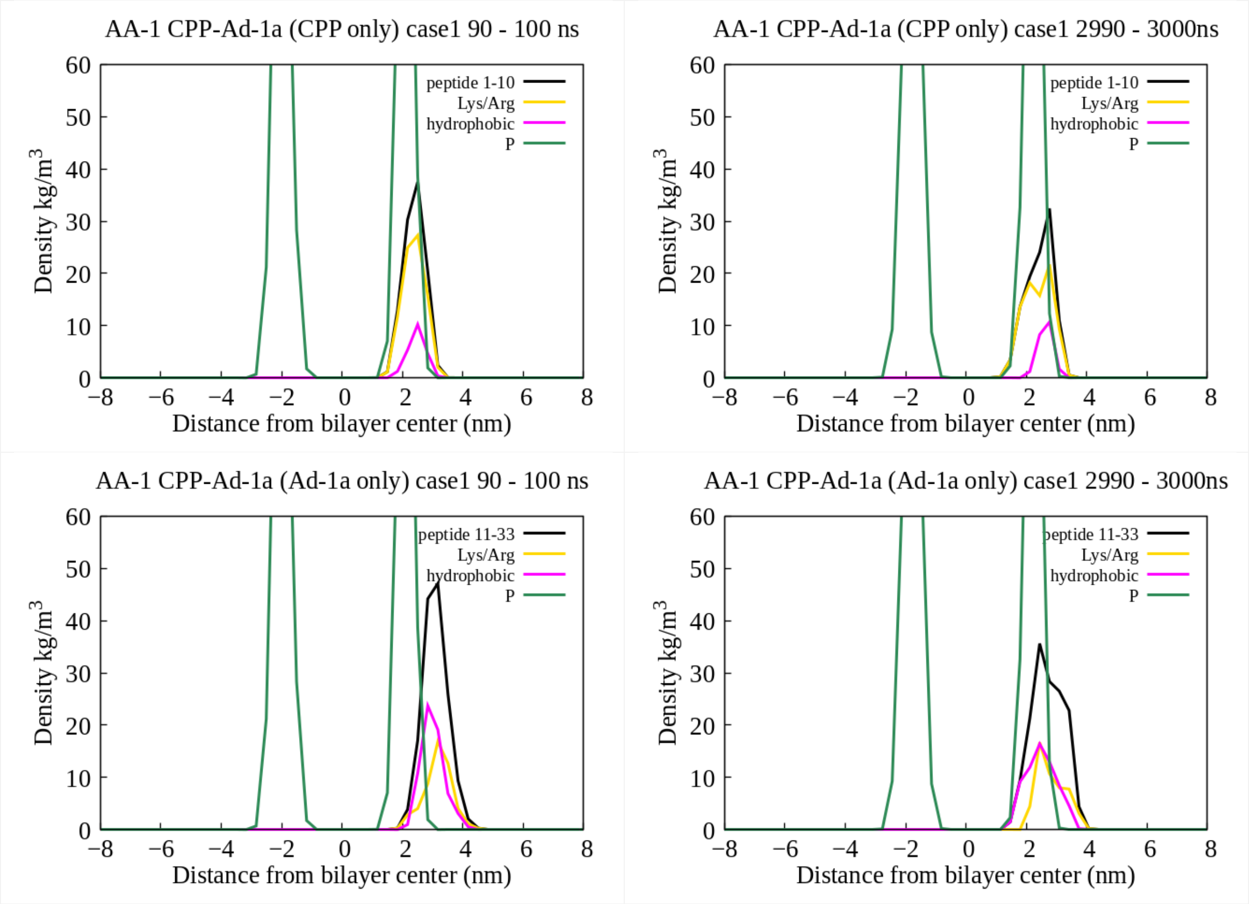

Figure S4b. Density profiles in AA-1 simulations (**case 1**) of CPP-Ad-1a peptide, averaged over denoted time intervals. **1st row**: CPP only (amino acids 1-10) **2nd row:** Ad-1a only (amino acids 11-33). See description in **Figure S4a**.

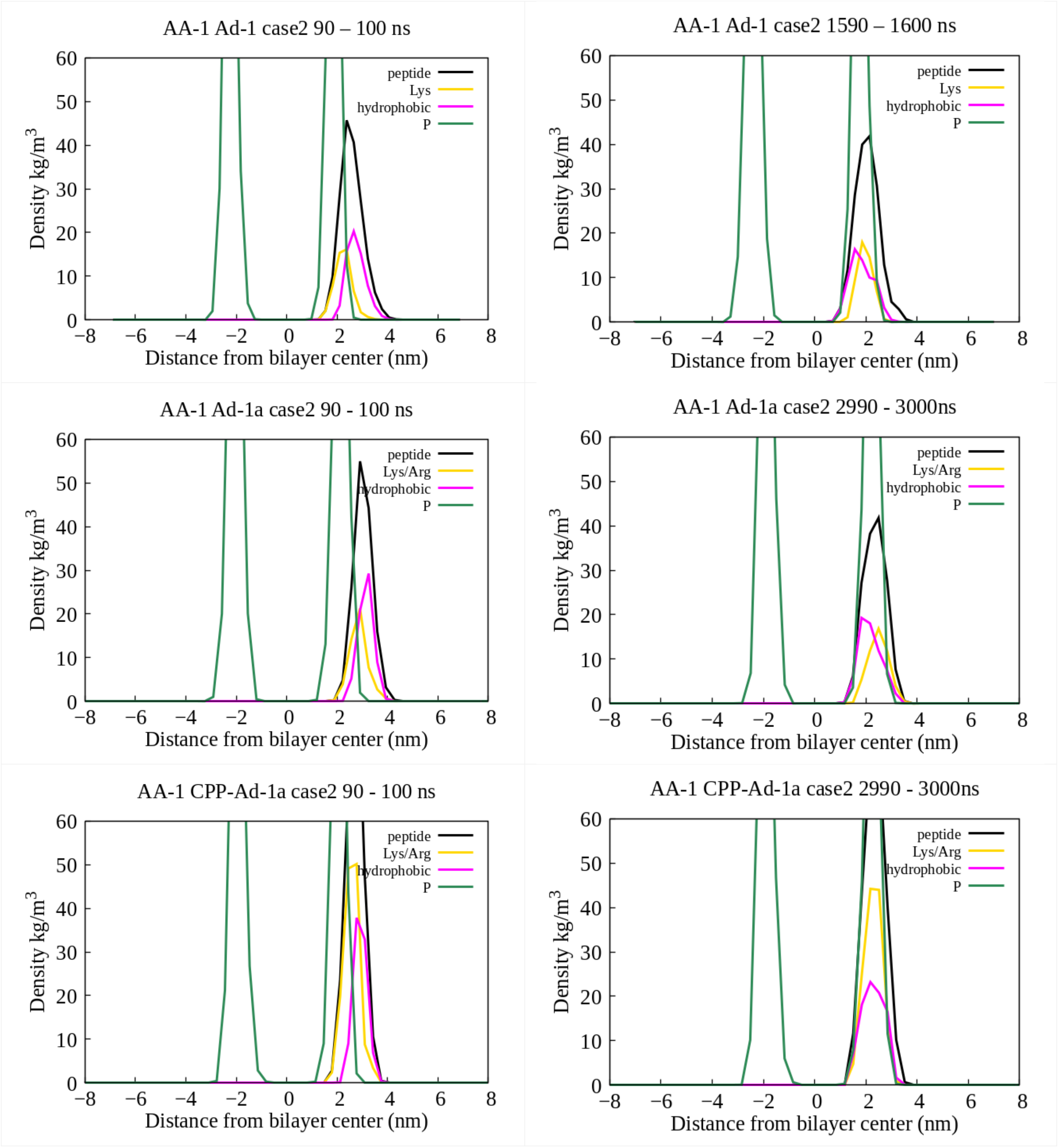

Figure S4c. Density profiles in AA-1 simulations (**case 2**), averaged over denoted time intervals. **1st row:** Ad-1, **2nd row:** Ad-1a, **3rd row:** CPP-Ad-1a. See description in **Figure S4a**.

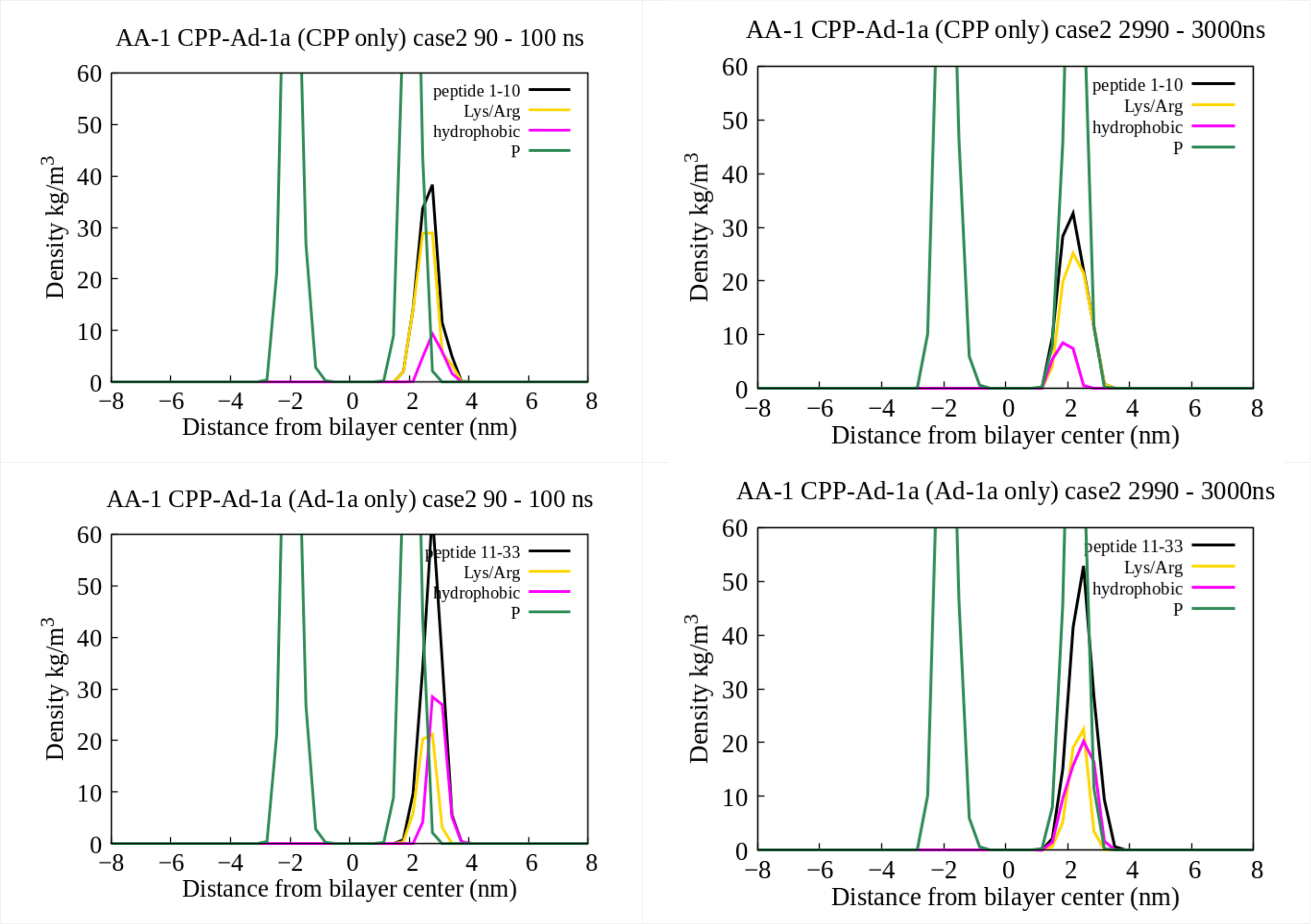

Figure S4d. Density profiles in AA-1 simulations (**case 2**) of CPP-Ad-1a peptide, averaged over denoted time intervals. **1st row**: CPP only (amino acids 1-10) **2nd row:** Ad-1a only (amino acids 11-33). See description in **Figure S4a**.

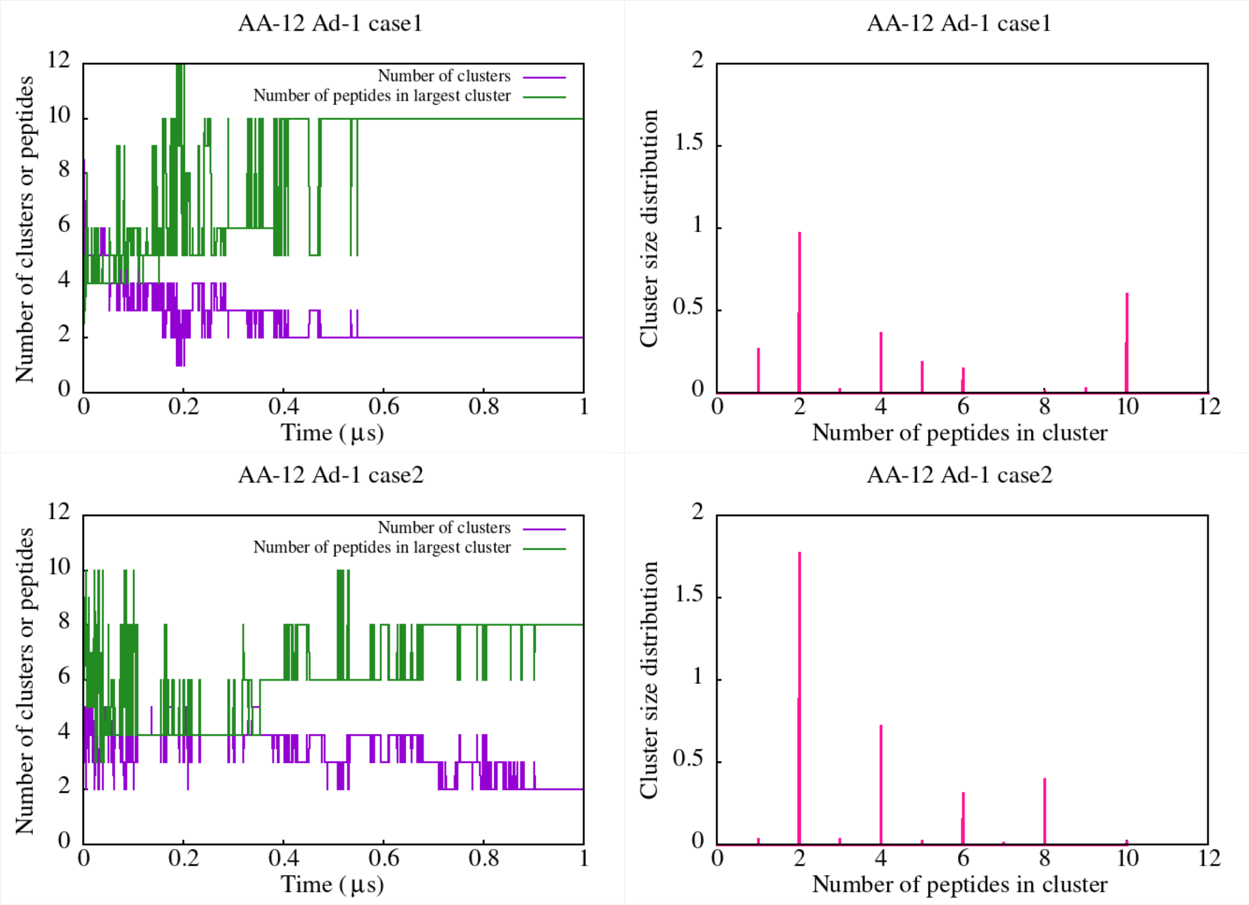

Figure S5a. Number of clusters, number of peptides in largest cluster and cluster size distribution in AA-12 simulations of Adepantin-1.

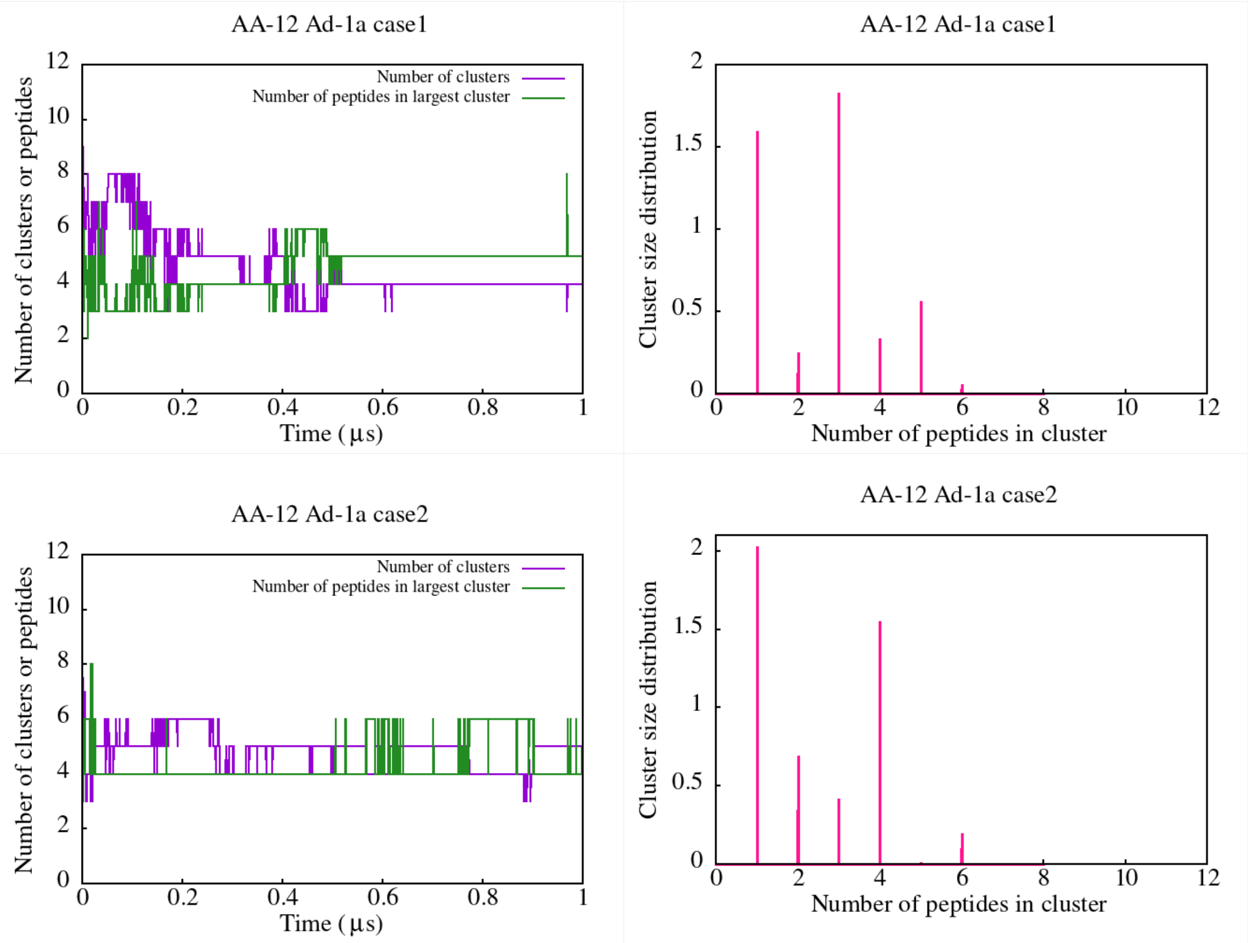

Figure S5b. Number of clusters, number of peptides in largest cluster and cluster size distribution in AA-12 simulations of Adepantin-1a.

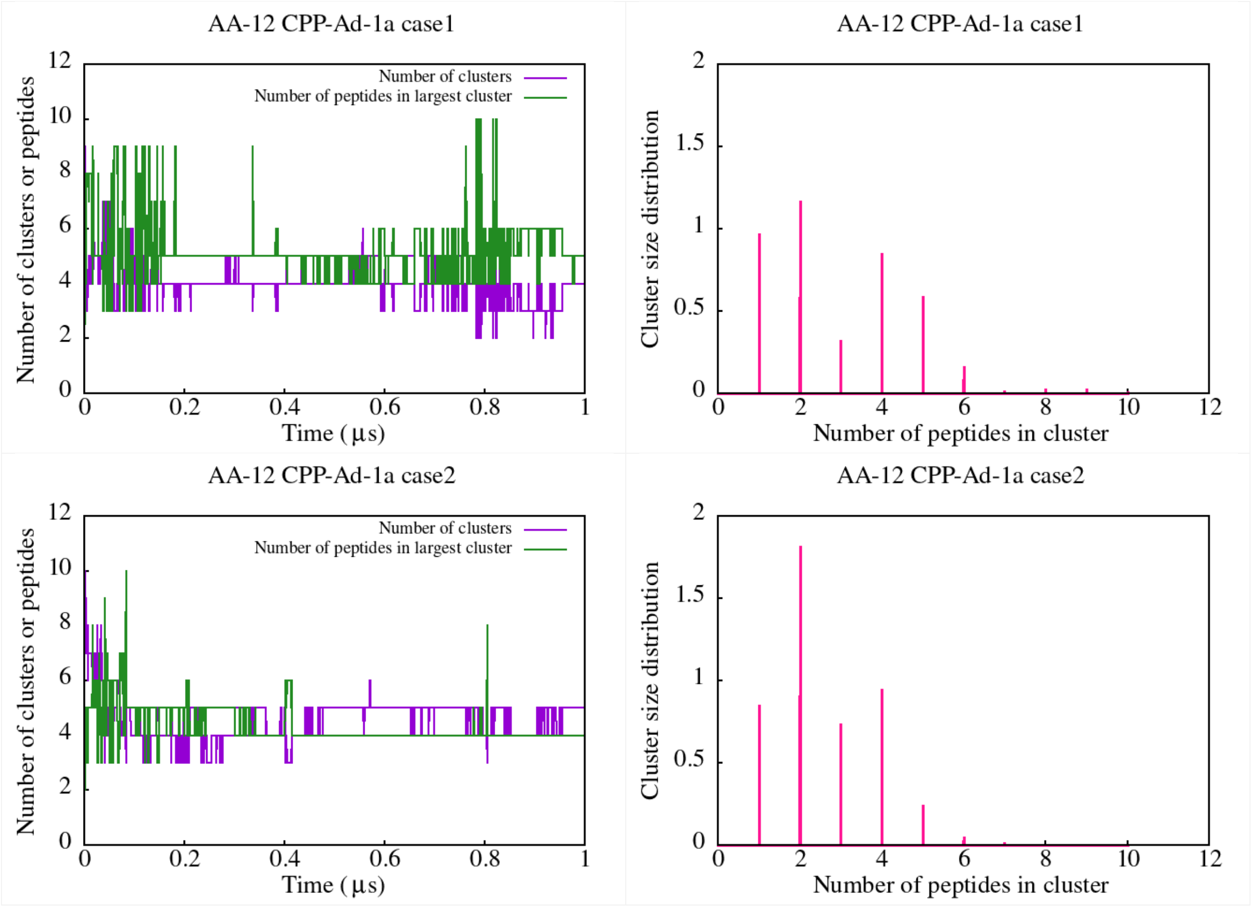

Figure S5c. Number of clusters, number of peptides in largest cluster and cluster size distribution in AA-12 simulations of CPP-Adepantin-1a.

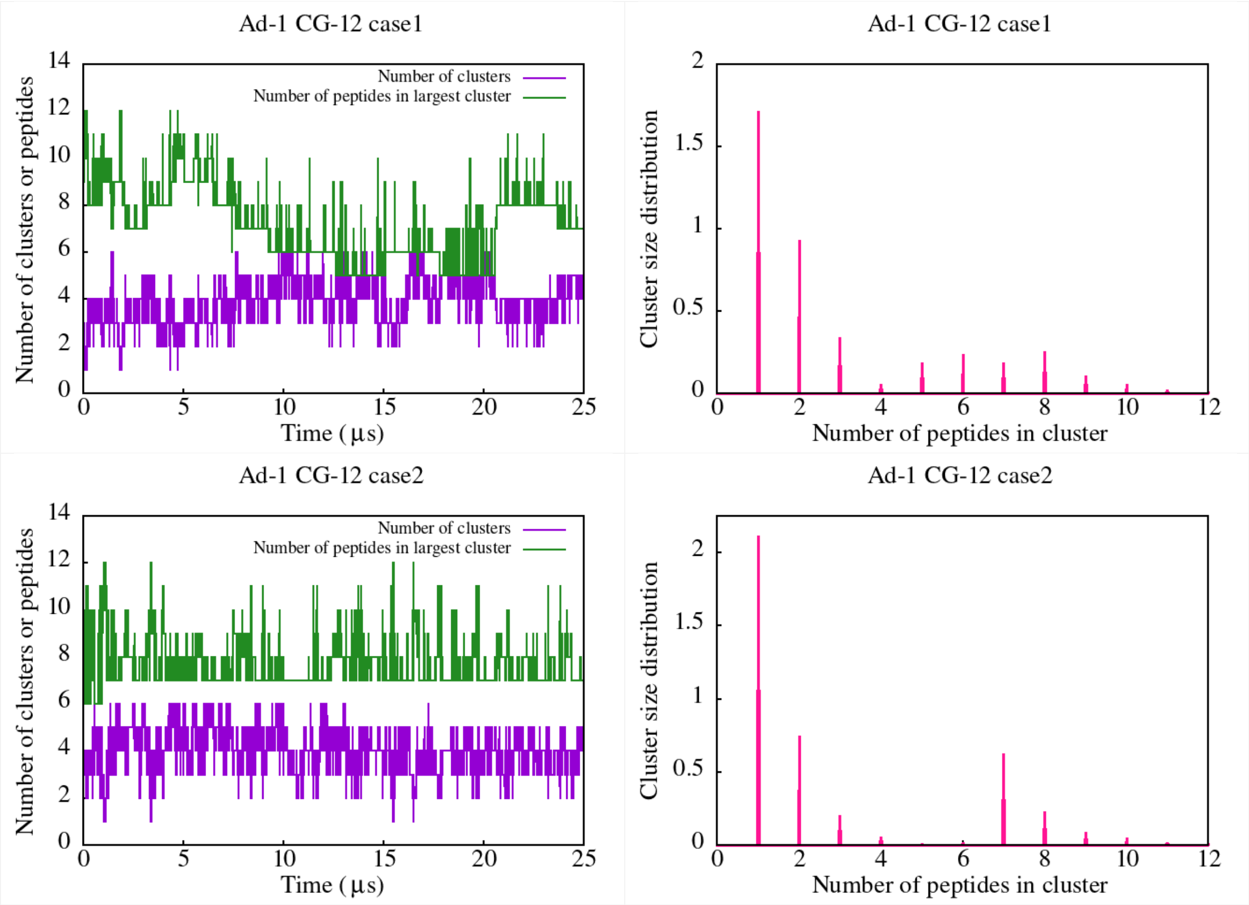

Figure S5d. Number of clusters, number of peptides in largest cluster and cluster size distribution in CG-12 simulations of Adepantin-1.

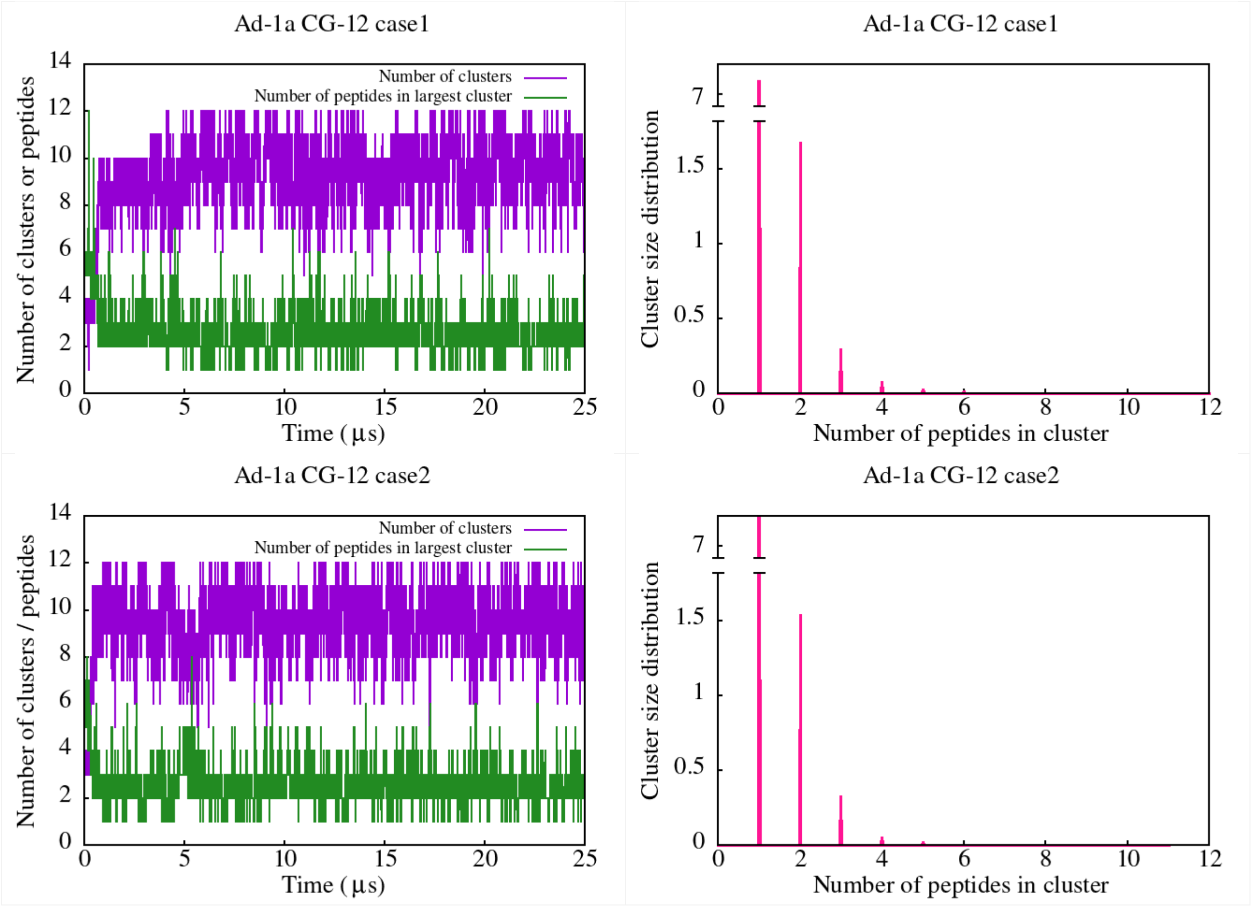

Figure S5e. Number of clusters, number of peptides in largest cluster and cluster size distribution in CG-12 simulations of Adepantin-1a.

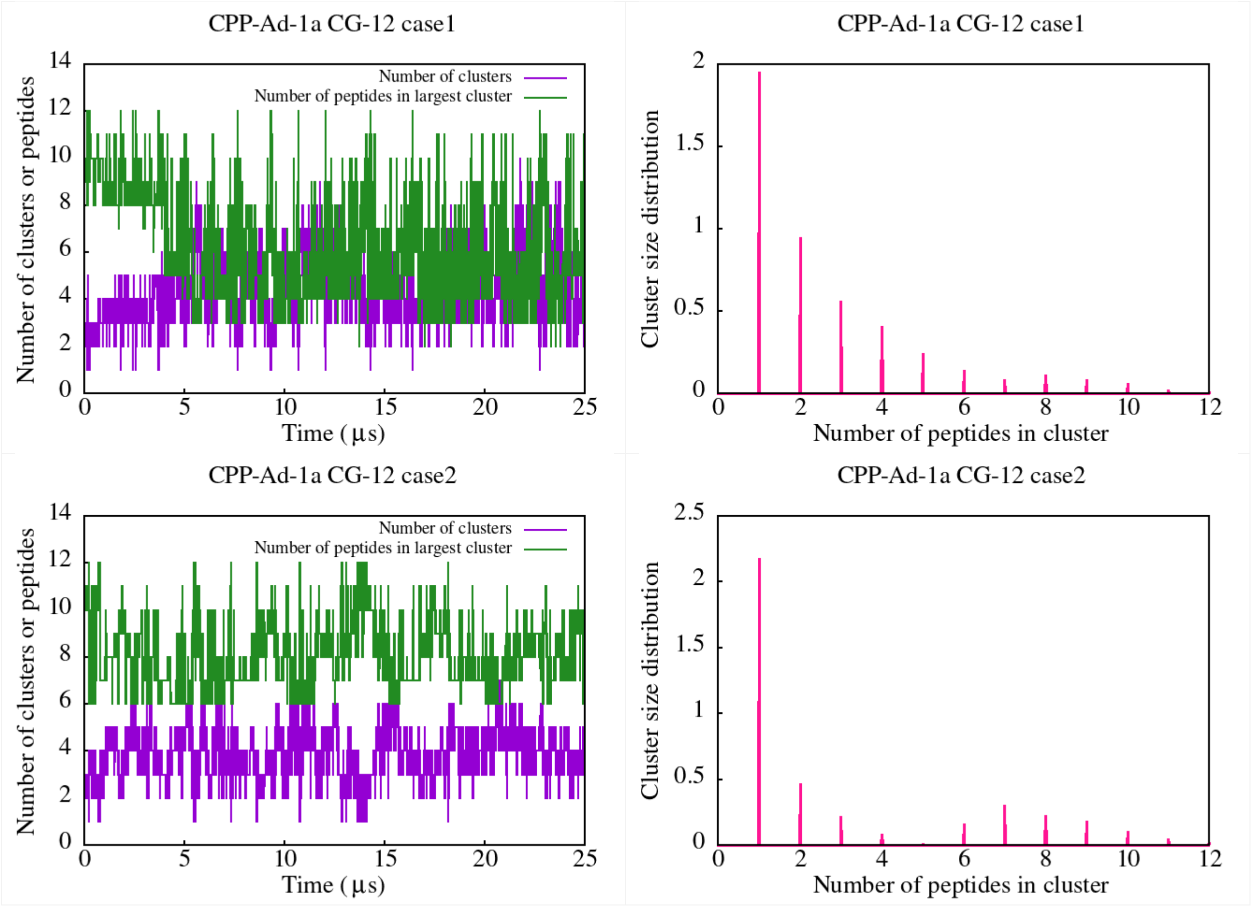

Figure S5f. Number of clusters, number of peptides in largest cluster and cluster size distribution in CG-12 simulations of CPP-Adepantin-1a.

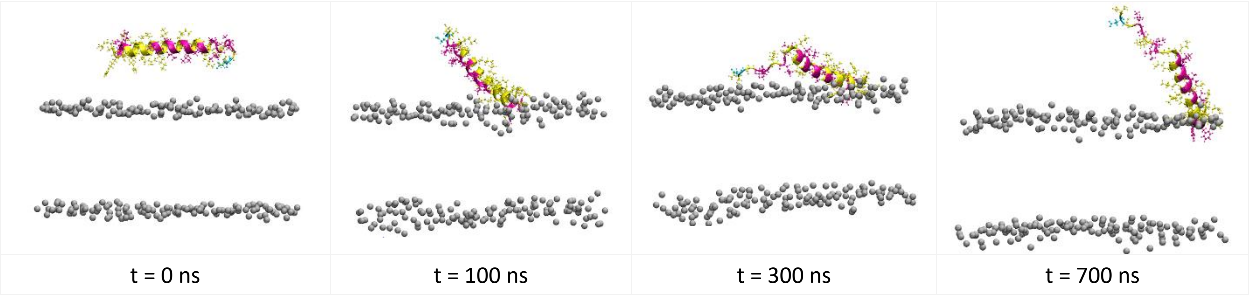

Figure S6a. Characteristic states during interaction of CPP-Ad-1a peptide with POPC membrane in AA-1 simulation.

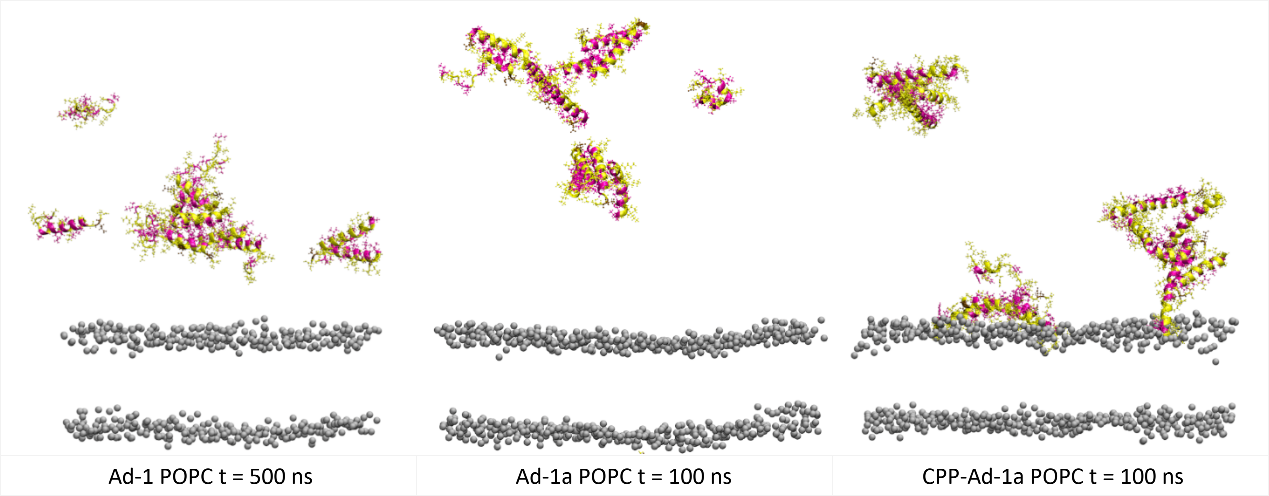

Figure S6b. Last states of interaction of 12 peptides with POPC membrane in AA-12 simulations. It is observed that peptides Ad-1 and Ad-1a do not bind to the neutral POPC membrane, unlike CPP-Ad-1a, which shows a certain tendency for binding. Compare with **Figure S2c**.

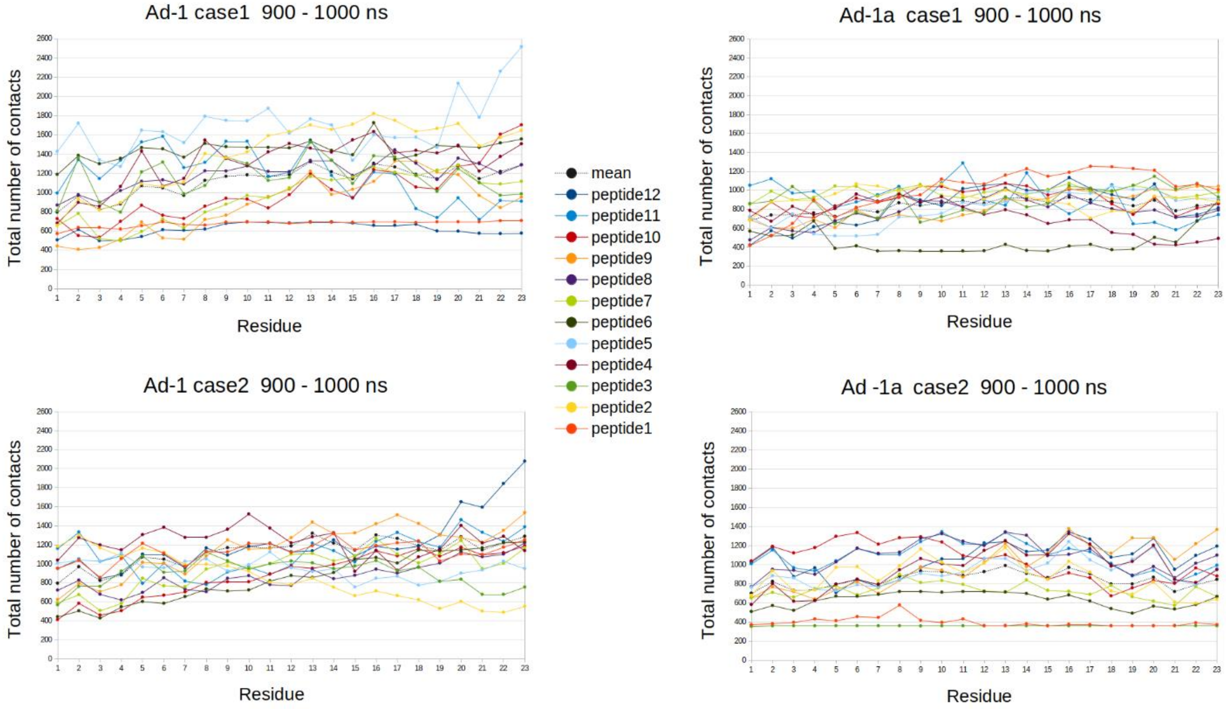

Figure S7. Total number of contacts for each residue with other residues, calculated over the final 100 ns of simulations involving 12 peptides. **Left**: Results from AA-12 Ad-1, **right:** AA-12 Ad-1a.

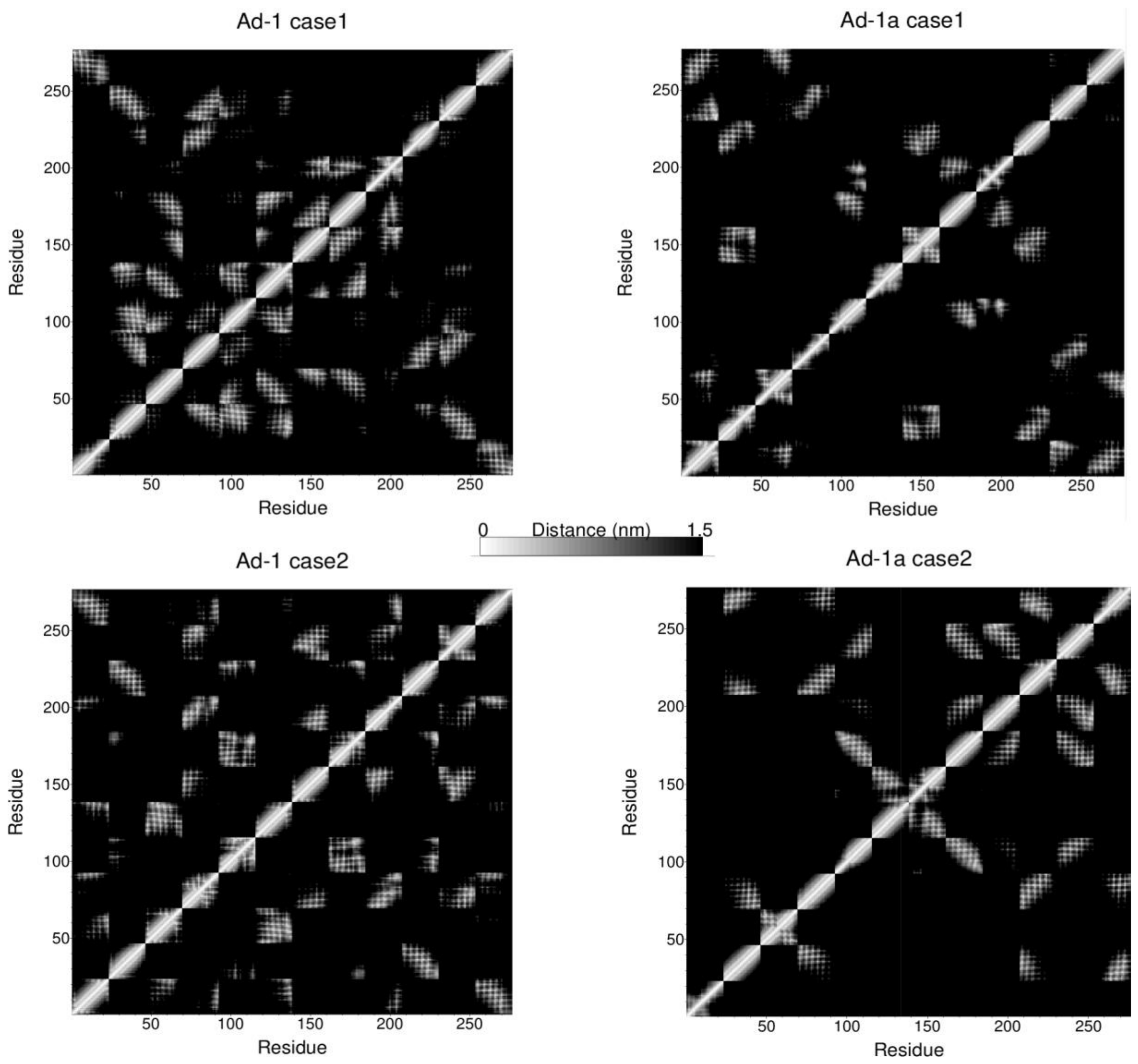

Figure S8. Distance matrices showing the smallest distances between residue pairs across all twelve peptides (a total of 23 * 12 = 276 residues), calculated over the final 100 ns of simulations. **Left**: Results from AA-12 Ad-1, **right:** AA-12 Ad-1a.

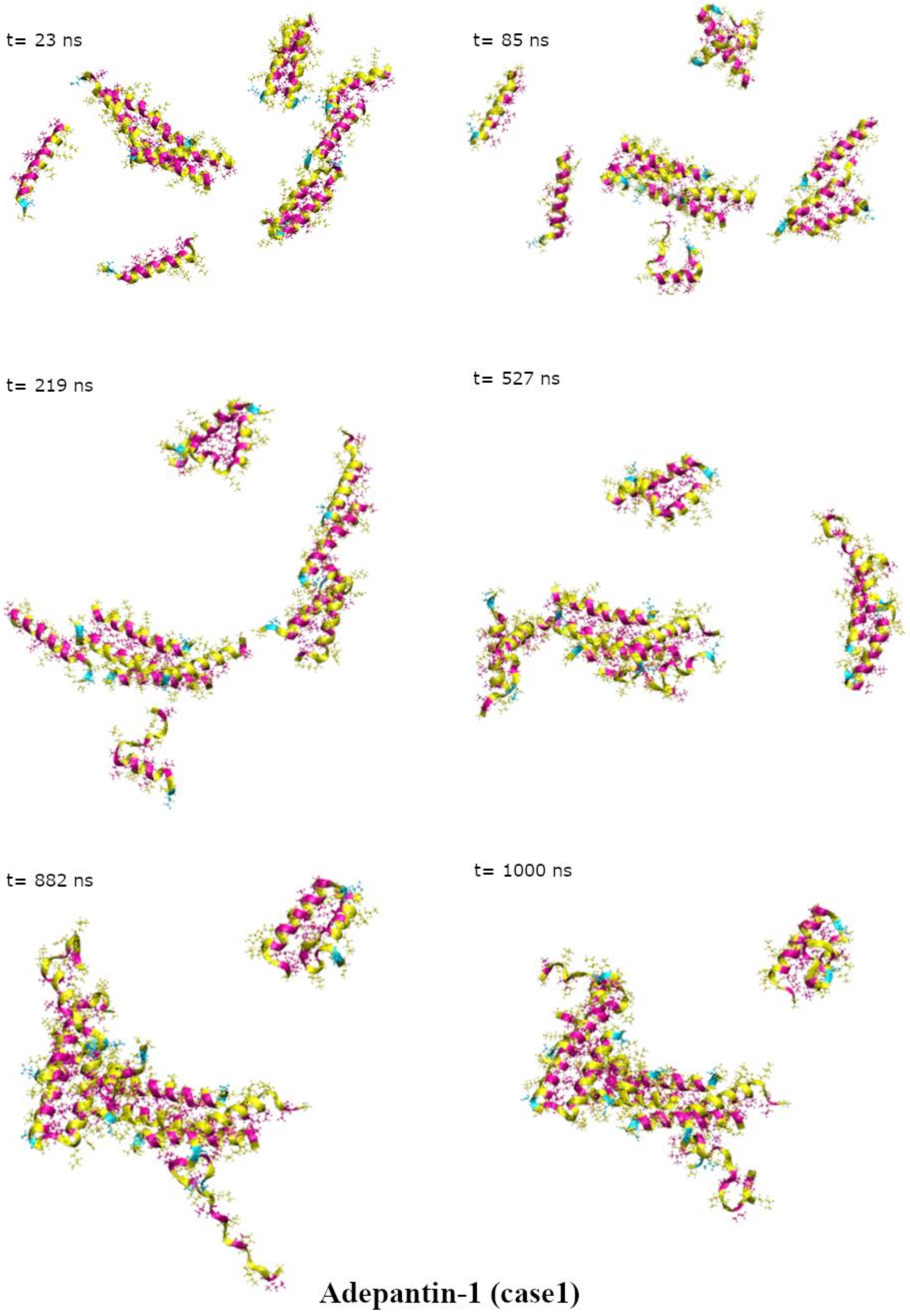

Figure S9a. Visual representation of the clustering of **Ad-1** in AA-12 simulation case1. Ad-1 shows a greater tendency to form larger clusters when binding to the POPE:POPG membrane compared to Ad-1a peptides (Compare with **Figure S9b**).

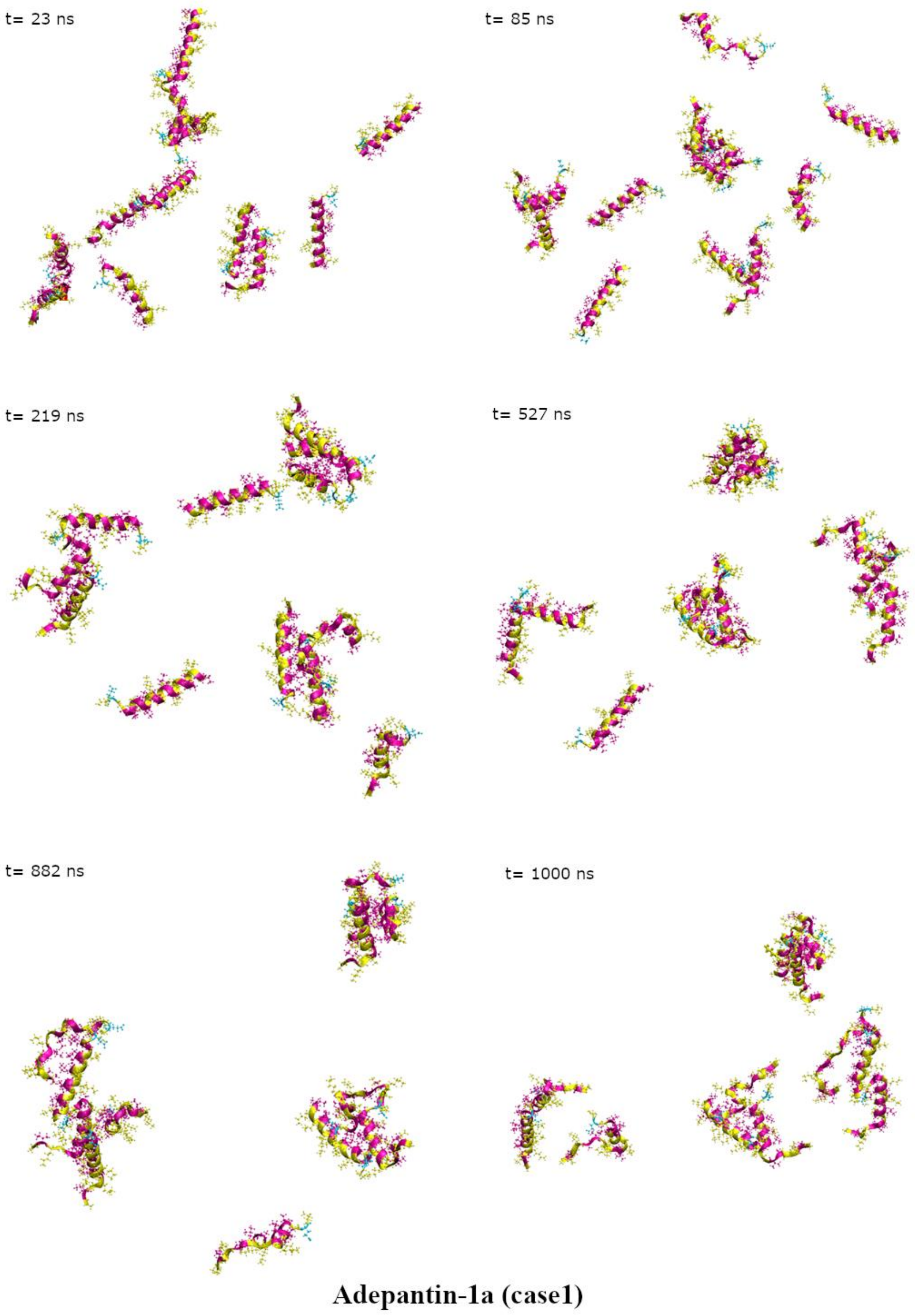

Figure S9b. Visual representation of the clustering of **Ad-1a** in AA-12 simulation case1. Ad-1a shows a tendency to form more smaller clusters when binding to the POPE:POPG membrane compared to Ad-1 peptides (Compare with **Figure S9a**).

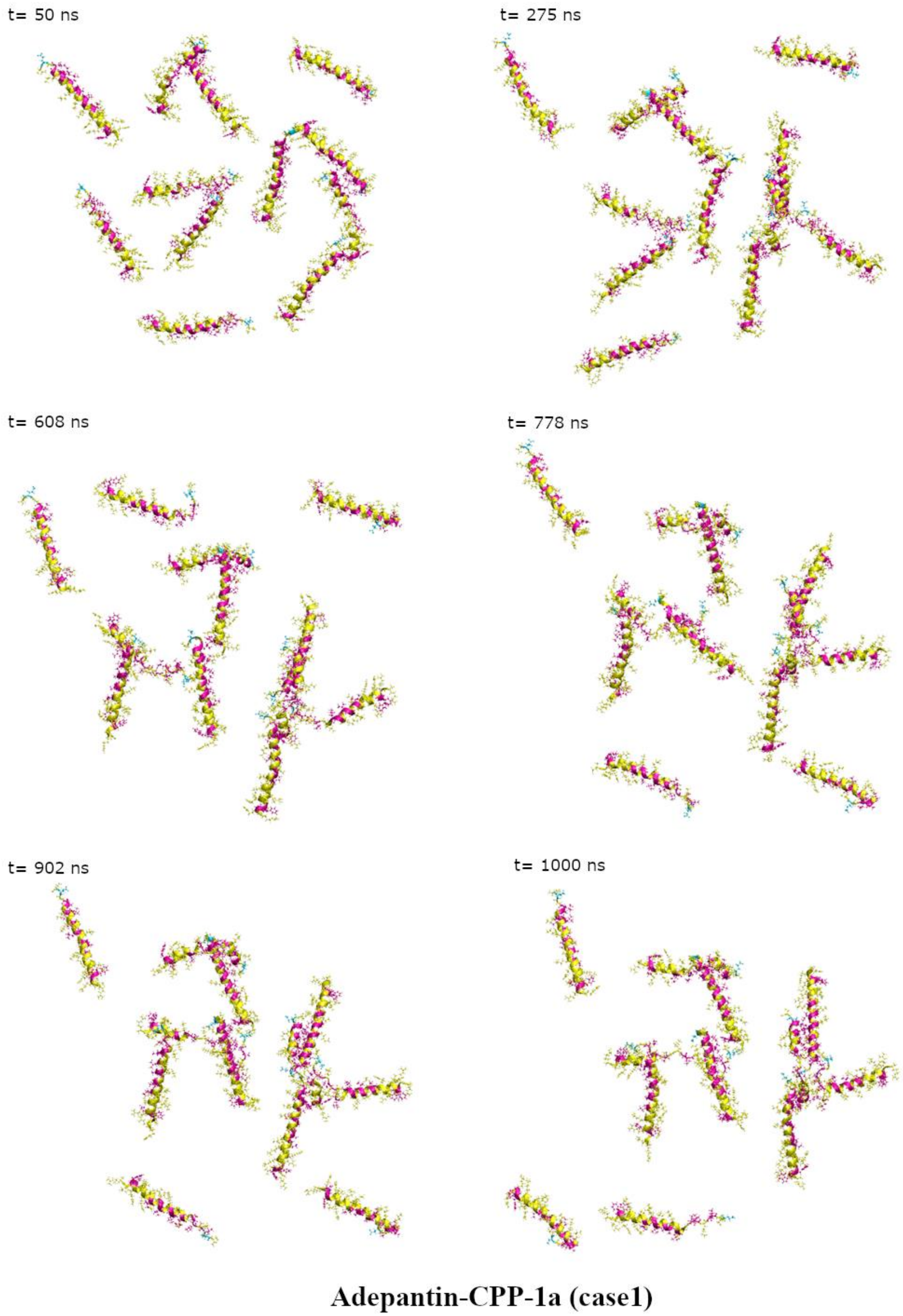

Figure S9c. Visual representation of the clustering of **CPP-Ad-1a** in AA-12 simulations case1. Clusters formed by CPP-Ad-1a peptides have a different spatial distribution and are not as dense as those of Ad-1 and Ad-1a peptides.

Table S1. 3D Hydrophobic moments in simulations AA-1 case1.

	Abs value of 3DHM vector (ÅkT/e)				Angle between 3DHM vector and +z axis (°)*			
**Diel. const**	**78.5**	**20.0**	**20.0**	**20.0**	**78.5**	**20.0**	**20.0**	**20.0**
**Timestamp (ns)**	**0**	**100**	**600**	**1000**	**0**	**100**	**600**	**1000**
Ad-1	15.6	60.3	35.4	29.0	82.4	50.0	37.7	112.0
**Timestamp (ns)**	**0**	**500**	**1600**	**3000**	**0**	**500**	**1600**	**3000**
Ad-1a	19.5	75.1	52.4	48.7	96.3	69.1	20.0	93.8
**Timestamp (ns)**	**0**	**100**	**400**	**3000**	**0**	**100**	**400**	**3000**
CPP-Ad-1a**	49.9	147.8	81.2	81.9	89.7	70.9	71.7	69.8
1-10**	8.7	-	-	44.3	59.4	-	-	13.5
11-33**	12.8	-	-	22.8	145.0	-	-	107.3

*If the angle is greater than 90 degrees, the vector is oriented towards the membrane.

** The last three rows' results are from the same simulation AA-1 CPP-Ad-1a. 1-10 refers to the first ten amino acids (CPP part), and 11-33 refers to the remaining part of the peptide CPP-Ad-1a.

Table S2. Electrostatic dipole moment vectors for the configurations shown in Fig 2 in the article.

	Abs value of electrostatic dipole moment vector (Debye)			
Diel. Const.	78.5	20.0	20.0	20.0
Timestamp	0 ns	100 ns	600 ns	1000 ns
**Ad-1**	5.7	23.4	18.0	13.1
Timestamp	0ns	500 ns	1600 ns	3000 ns
**Ad-1a**	5.5	21.2	15.7	15.8
**Timestamp**	0 ns	100 ns	400 ns	3000 ns
**CPP-Ad-1a*** **	11.4	28.9	14.9	13.4
**1-10*** **	1.7	8.4	7.9	9.0
**11-33*** **	5.5	10.0	6.8	7.5

*** Last three rows results are from the same simulation AA-1 CPP-Ad1a. 1-10 refers to the first ten amino acids (CPP part), 11-33 refers to the remaining part of the peptide CPP-Ad1a.

Bibliography:

1. Scorciapino, M. Antimicrobial Peptidomimetics: Reinterpreting Nature to Deliver Innovative Therapeutics. *Front Immunol* 2012, *3*, doi:10.3389/fimmu.2012.00171.

2. Mojsoska, B.; Jenssen, H. Peptides and Peptidomimetics for Antimicrobial Drug Design. *Pharmaceuticals* 2015, *8*, 366–415, doi:10.3390/ph8030366.

3. Spänig, S.; Heider, D. Encodings and Models for Antimicrobial Peptide Classification for Multi-Resistant Pathogens. *BioData Min* 2019, *12*, 7, doi:10.1186/s13040-019-0196-x.

4. Juretić, D.; Vukičević, D.; Ilić, N.; Antcheva, N.; Tossi, A. Computational Design of Highly Selective Antimicrobial Peptides. *J Chem Inf Model* 2009, *49*, 2873–2882, doi:10.1021/ci900327a.

5. Robles-Loaiza, A.A.; Pinos-Tamayo, E.A.; Mendes, B.; Ortega-Pila, J.A.; Proaño-Bolaños, C.; Plisson, F.; Teixeira, C.; Gomes, P.; Almeida, J.R. Traditional and Computational Screening of Non-Toxic Peptides and Approaches to Improving Selectivity. *Pharmaceuticals* 2022, *15*, 323, doi:10.3390/ph15030323.

6. Juretić, D.; Vukičević, D.; Petrov, D.; Novković, M.; Bojović, V.; Lučić, B.; Ilić, N.; Tossi, A. Knowledge-Based Computational Methods for Identifying or Designing Novel, Non-Homologous Antimicrobial Peptides. *European Biophysics Journal* 2011, *40*, 371–385, doi:10.1007/s00249-011-0674-7.

7. Juretić, D.; Vukičević, D.; Tossi, A. Tools for Designing Amphipathic Helical Antimicrobial Peptides. *Methods Mol Biol* 2017, *1548*, 23–34, doi:10.1007/978-1-4939-6737-7_2.

8. Kamech, N.; Vukičević, D.; Ladram, A.; Piesse, C.; Vasseur, J.; Bojović, V.; Simunić, J.; Juretić, D. Improving the Selectivity of Antimicrobial Peptides from Anuran Skin. *J Chem Inf Model* 2012, *52*, 3341–3351, doi:10.1021/ci300328y.

9. Juretić, D.; Zoranić, L.; Zucić, D. Basic Charge Clusters and Predictions of Membrane Protein Topology. *J Chem Inf Comput Sci* 2002, *42*, 620–632, doi:10.1021/ci010263s.

10. Preußke, N.; Sönnichsen, F.D.; Leippe, M. A Guided Tour through α-Helical Peptide Antibiotics and Their Targets. *Biosci Rep* 2023, *43*, doi:10.1042/BSR20230474.

11. Thankappan, B.; Jeyarajan, S.; Hiroaki, S.; Anbarasu, K.; Natarajaseenivasan, K.; Fujii, N. Antimicrobial and Antibiofilm Activity of Designed and Synthesized Antimicrobial Peptide, KABT-AMP. *Appl Biochem Biotechnol* 2013, *170*, 1184–1193, doi:10.1007/S12010-013-0258-3.

12. Timmons, P.B.; Hewage, C.M. ENNAVIA Is a Novel Method Which Employs Neural Networks for Antiviral and Anti-Coronavirus Activity Prediction for Therapeutic Peptides. *Brief Bioinform* 2021, *22*, doi:10.1093/BIB/BBAB258.

13. Timmons, P.B.; Hewage, C.M. HAPPENN Is a Novel Tool for Hemolytic Activity Prediction for Therapeutic Peptides Which Employs Neural Networks. *Scientific Reports 2020 10:1* 2020, *10*, 1–18, doi:10.1038/s41598-020-67701-3.

14. Timmons, P.B.; Hewage, C.M. ENNAACT Is a Novel Tool Which Employs Neural Networks for Anticancer Activity Classification for Therapeutic Peptides. *Biomedicine & Pharmacotherapy* 2021, *133*, 111051, doi:10.1016/j.biopha.2020.111051.

15. Novković, M.; Simunić, J.; Bojović, V.; Tossi, A.; Juretić, D. DADP: The Database of Anuran Defense Peptides. *Bioinformatics* 2012, *28*, 1406–1407, doi:10.1093/BIOINFORMATICS/BTS141.

16. Manavalan, B.; Patra, M.C. MLCPP 2.0: An Updated Cell-Penetrating Peptides and Their Uptake Efficiency Predictor. *J Mol Biol* 2022, *434*, 167604, doi:10.1016/J.JMB.2022.167604.

17. Gawde, U.; Chakraborty, S.; Waghu, F.H.; Barai, R.S.; Khanderkar, A.; Indraguru, R.; Shirsat, T.; Idicula-Thomas, S. CAMPR4: A Database of Natural and Synthetic Antimicrobial Peptides. *Nucleic Acids Res* 2023, *51*, D377, doi:10.1093/NAR/GKAC933.

18. Burdukiewicz, M.; Sidorczuk, K.; Rafacz, D.; Pietluch, F.; Chilimoniuk, J.; Rödiger, S.; Gagat, P. Proteomic Screening for Prediction and Design of Antimicrobial Peptides with AmpGram. *Int J Mol Sci* 2020, *21*, 1–13, doi:10.3390/IJMS21124310.

19. Schaduangrat, N.; Nantasenamat, C.; Prachayasittikul, V.; Shoombuatong, W. ACPred: A Computational Tool for the Prediction and Analysis of Anticancer Peptides. *Molecules* 2019, *24*, doi:10.3390/MOLECULES24101973.

20. Boopathi, V.; Subramaniyam, S.; Malik, A.; Lee, G.; Manavalan, B.; Yang, D.C. MACPpred: A Support Vector Machine-Based Meta-Predictor for Identification of Anticancer Peptides. *Int J Mol Sci* 2019, *20*, doi:10.3390/IJMS20081964.

21. Sangaraju, V.K.; Pham, N.T.; Wei, L.; Yu, X.; Manavalan, B. MACPpred 2.0: Stacked Deep Learning for Anticancer Peptide Prediction with Integrated Spatial and Probabilistic Feature Representations. *J Mol Biol* 2024, doi:10.1016/J.JMB.2024.168687.

22. Chowdhury, A.S.; Reehl, S.M.; Kehn-Hall, K.; Bishop, B.; Webb-Robertson, B.J.M. Better Understanding and Prediction of Antiviral Peptides through Primary and Secondary Structure Feature Importance. *Sci Rep* 2020, *10*, doi:10.1038/s41598-020-76161-8.

23. Schaduangrat, N.; Nantasenamat, C.; Prachayasittikul, V.; Shoombuatong, W. Meta-IAVP: A Sequence-Based Meta-Predictor for Improving the Prediction of Antiviral Peptides Using Effective Feature Representation. *Int J Mol Sci* 2019, *20*, doi:10.3390/IJMS20225743.

24. Meher, P.K.; Sahu, T.K.; Saini, V.; Rao, A.R. Predicting Antimicrobial Peptides with Improved Accuracy by Incorporating the Compositional, Physico-Chemical and Structural Features into Chou's General PseAAC. *Scientific Reports 2017 7:1* 2017, *7*, 1–12, doi:10.1038/srep42362.

25. Zhang, J.; Sun, X.; Zhao, H.; Zhou, X.; Zhang, Y.; Xie, F.; Li, B.; Guo, G. In Silico Design and Synthesis of Antifungal Peptides Guided by Quantitative Antifungal Activity. *J Chem Inf Model* 2024, *64*, 4277–4285, doi:10.1021/ACS.JCIM.4C00142.

26. Zhang, J.; Yang, L.; Tian, Z.; Zhao, W.; Sun, C.; Zhu, L.; Huang, M.; Guo, G.; Liang, G. Large-Scale Screening of Antifungal Peptides Based on Quantitative Structure-Activity Relationship. *ACS Med Chem Lett* 2022, *13*, 99–104, doi:10.1021/ACSMEDCHEMLETT.1C00556.

27. Manavalan, B.; Shin, T.H.; Kim, M.O.; Lee, G. AIPpred: Sequence-Based Prediction of Anti-Inflammatory Peptides Using Random Forest. *Front Pharmacol* 2018, *9*, doi:10.3389/FPHAR.2018.00276/FULL.

28. Khatun, M.S.; Hasan, M.M.; Kurata, H. PreAIP: Computational Prediction of Anti-Inflammatory Peptides by Integrating Multiple Complementary Features. *Front Genet* 2019, *10*, 414602, doi:10.3389/FGENE.2019.00129/BIBTEX.

29. Gupta, S.; Sharma, A.K.; Shastri, V.; Madhu, M.K.; Sharma, V.K. Prediction of Anti-Inflammatory Proteins/Peptides: An Insilico Approach. *J Transl Med* 2017, *15*, 1–11, doi:10.1186/S12967-016-1103-6/TABLES/3.

30. Gupta, S.; Kapoor, P.; Chaudhary, K.; Gautam, A.; Kumar, R.; Raghava, G.P.S. In Silico Approach for Predicting Toxicity of Peptides and Proteins. *PLoS One* 2013, *8*, doi:10.1371/JOURNAL.PONE.0073957.

31. Gupta, S.; Kapoor, P.; Chaudhary, K.; Gautam, A.; Kumar, R.; Raghava, G.P.S. Peptide Toxicity Prediction. *Methods Mol Biol* 2015, *1268*, 143–157, doi:10.1007/978-1-4939-2285-7_7.

32. Juretić, D.; Lučin, A. The Preference Functions Method for Predicting Protein Helical Turns with Membrane Propensity. *J Chem Inf Comput Sci* 1998, *38*, 575–585, doi:https://doi.org/10.1021/ci970073a.

33. Hu, G.; Katuwawala, A.; Wang, K.; Wu, Z.; Ghadermarzi, S.; Gao, J.; Kurgan, L. flDPnn: Accurate Intrinsic Disorder Prediction with Putative Propensities of Disorder Functions. *Nature Communications 2021 12:1* 2021, *12*, 1–8, doi:10.1038/s41467-021-24773-7.

34. Kauffman, W.B.; Guha, S.; Wimley, W.C. Synthetic Molecular Evolution of Hybrid Cell Penetrating Peptides. *Nature Communications 2018 9:1* 2018, *9*, 1–10, doi:10.1038/s41467-018-04874-6.

35. Buccini, D.F.; Cardoso, M.H.; Franco, O.L. Antimicrobial Peptides and Cell-Penetrating Peptides for Treating Intracellular Bacterial Infections. *Front Cell Infect Microbiol* 2021, *10*, 612931, doi:10.3389/FCIMB.2020.612931/BIBTEX.

36. Wang, C.; Yang, Y.; Cao, Y.; Liu, K.; Shi, H.; Guo, X.; Liu, W.; Hao, R.; Song, H.; Zhao, R. Nanocarriers for the Delivery of Antibiotics into Cells against Intracellular Bacterial Infection. *Biomater Sci* 2023, *11*, 432–444, doi:10.1039/D2BM01489K.

37. Joosten, R.P.; Te Beek, T.A.H.; Krieger, E.; Hekkelman, M.L.; Hooft, R.W.W.; Schneider, R.; Sander, C.; Vriend, G. A Series of PDB Related Databases for Everyday Needs. *Nucleic Acids Res* 2015, *43*, D364–D368, doi:10.1093/NAR/GKQ1105.

38. Kabsch, W.; Sander, C. Dictionary of Protein Secondary Structure: Pattern Recognition of Hydrogen-Bonded and Geometrical Features. *Biopolymers* 1983, *22*, 2577–2637, doi:10.1002/BIP.360221211.
